# Epidemiologic patterns of biliary tract cancer in the United States: 2001–2015

**DOI:** 10.1186/s12885-022-10286-z

**Published:** 2022-11-16

**Authors:** Jill Koshiol, Binbing Yu, Shaum M. Kabadi, Katherine Baria, Rachna T. Shroff

**Affiliations:** 1 Division of Cancer Epidemiology and Genetics, National Cancer Institute, Bethesda, MD USA; 2 Oncology Biometrics, AstraZeneca Pharmaceuticals, Gaithersburg, MD USA; 3 Medical Affairs Epidemiology and Real-World Evidence, AstraZeneca Pharmaceuticals, Gaithersburg, MD USA; 4 Global Medical Affairs, AstraZeneca Pharmaceuticals, Gaithersburg, MD USA; 5 Division of Hematology and Oncology, University of Arizona Cancer Center, 1515 N. Campbell Ave, PO Box 245024, Tucson, AZ 85724-5024 USA

**Keywords:** Biliary tract cancer, Intrahepatic cholangiocarcinoma, Extrahepatic cholangiocarcinoma, Gallbladder cancer, Ampulla of Vater cancer

## Abstract

**Background:**

Biliary tract cancer (BTC) includes intrahepatic cholangiocarcinoma (ICC), extrahepatic cholangiocarcinoma, gallbladder cancer, and ampulla of Vater cancer (AVC). Although BTC is rare in the US, incidence is increasing and elevated in certain populations. This study examined BTC epidemiology in the US by age, sex, race/ethnicity, geographic region, and anatomic site.

**Methods:**

BTC incidence, prevalence, mortality, and survival from 2001 to 2015 were evaluated using the National Cancer Institute’s Surveillance, Epidemiology, and End Results Program and the Centers for Disease Control and Prevention’s National Program of Cancer Registries databases. Incidence and mortality rates were calculated and reported as age-standardized rates. Data were assessed by age, anatomic sites, geographic region, and race/ethnicity, and a joinpoint regression model was used to predict trends for age-adjusted BTC incidence and mortality rates.

**Results:**

BTC incidence increased during the study period (annual percent change = 1.76, 95% confidence interval [1.59–1.92]), with the highest increase in ICC (6.65 [6.11–7.19]). Incidence of unspecified BTC initially increased but has recently begun to drop. Hispanic, Asian/Pacific Islander, Black, or American Indian/Alaska Native race/ethnicity was associated with higher BTC mortality rates than White race/ethnicity. Patients with ICC had the highest mortality rate (age-standardized rate = 1.87/100,000 person-years [1.85–1.88]). Five-year survival was 15.2% for all BTC, ranging from 8.5% (ICC) to 34.5% (AVC), and patients with distant disease at diagnosis had lower survival (3%) compared with those with regional (19.1%) or locally advanced disease (31.5%).

**Conclusions:**

BTC incidence increased, survival was low across all subtypes, and mortality was greatest in patients with ICC. This underscores the serious, increasing unmet need among patients with BTC. Treatment options are limited, although clinical studies investigating immunotherapy, targeted therapies, and alternative chemotherapy combinations are ongoing. Epidemiological insights may improve patient care and inform the integration of novel therapies for BTC.

**Supplementary Information:**

The online version contains supplementary material available at 10.1186/s12885-022-10286-z.

## Background

Biliary tract cancer (BTC) forms a heterogeneous group of hepatic and perihepatic tumors, including gallbladder cancer (GBC), cholangiocarcinoma (extrahepatic [ECC] and intrahepatic [ICC]), and ampulla of Vater cancer (AVC) [1]. In the US, liver and intrahepatic bile duct cancer are predicted to account for approximately 41,260 new cases in 2022, and GBC and other biliary cancers for approximately 12,130 new diagnoses in 2022 [2]. In the US, American Indian and Hispanic individuals are at greater risk for BTC, constituting an increased public health burden in these populations [3–5]. Late diagnosis, poor prognosis, and limited treatment options also increase disease burden related to BTC [1, 6, 7]. The most definitive BTC therapy includes surgical resection; however, only around 20% of tumors are considered resectable as most cases present at an advanced stage, and, as resection alone confers a high rate of recurrence, adjuvant chemotherapy and/or radiotherapy is needed to improve relapse-free and overall survival rates [8]. Advanced cancer treatment includes primarily chemotherapy, though areas of research interest include liver transplant, liver-directed therapies, immunotherapy, and targeted therapy [9–15]. Recently, durvalumab, an anti-PD-L1 antibody, in combination with gemcitabine and cisplatin was approved by the US Food and Drug Administration for treatment of individuals with advanced BTC, based on the results of the global, randomized, Phase III TOPAZ-1 study [16, 17].

Epidemiologic research for BTC is often limited to one anatomic site (e.g. ICC) or does not differentiate BTC from liver cancer [5, 7, 18–20], making it difficult to extract relevant information about this cancer group. BTC subtypes have distinct risk factors, prognoses, and likely respond to treatment differently, so monitoring the epidemiology of BTC and its anatomic sites will inform clinical program development and research.

There is an emerging interest in BTC because of the discovery of targetable genetic alterations [21], particularly for ICC tumors [22], while a smaller proportion of ECCs and GBCs have such mutations [23]. BTC molecular characterization studies are ongoing [24]. Molecular changes provide potential new therapy targets and may inform etiology. Understanding descriptive patterns of BTC incidence, survival, mortality, and prevalence can complement emerging insight into the molecular basis of these tumors, generating etiologic hypotheses and identifying gaps in clinical care.

US trends in BTC incidence, prevalence, mortality, and survival by age, sex, anatomic site, geographic region (state), and race/ethnicity were analyzed in this study. Real-world overall BTC rates are reported for comparison with other studies [4, 25, 26], as well as rates for each anatomic site, to identify unique epidemiological features for these disparate cancer types.

## Methods

The aim of this study was to describe BTC epidemiology and identify unique epidemiological features for BTC subtypes to improve patient care and inform the integration of novel therapies for BTC.

### Statistical analysis

BTC incidence and mortality data from the 2001–2015 National Program of Cancer Registries-Surveillance, Epidemiology, and End Results (NPCR-SEER) database [27], mortality data from the SEER 18 Incidence-based mortality database [28], and BTC prevalence and survival data from the 2001–2015 SEER 18 database were evaluated [29]. Mortality rates for BTC, GBC, and ICC were evaluated from the NPCR-SEER database while mortality rates for ECC, AVC, and overlapping/not otherwise specified (Overlapping & NOS) were taken from the SEER 18 Incidence-based mortality database. The NPCR supports central cancer registries in 46 states, the District of Columbia, Puerto Rico, the US Pacific Island Jurisdictions, and the US Virgin Islands, representing 97% of the US population [30]. The SEER 18 database comprises 18 registries representing approximately 28% of the US population based on the US 2010 census. Although a few states are funded both by CDC as NPCR registries and NCI as SEER registries, each state only contributed data once for each analysis. The Supplementary Methods (Additional File 1) includes additional details of the data sources, cancer classifications and excluded lymphohematologic tumors (Supplementary Table 1, Additional File 1).

The analysis included a defined population of patients aged ≥ 20 years who were diagnosed with BTC as a primary cancer during 2001–2015. BTC incidence rate was defined as the number of new cases of BTC divided by the number of persons at risk for BTC during a given year. Mortality rate was defined as the number of BTC-related deaths divided by the total population for a given year. BTC incidence and mortality rates were reported as age-standardized rates (ASRs; per 100,000 person-years) using the 2000 US standard population. Five-year relative survival for patients with BTC was defined as the percentage of patients who survived the effects of their cancer for 5 years, excluding the risk of dying from other causes. BTC prevalence, a function of past incidence and survival, was defined as the number of patients alive as of January 1, 2015, who were diagnosed with BTC between January 1, 2010 and January 1, 2015, and was reported per 100,000 person-years.

Data were assessed by age (20–29, 30–39, 40–49, 50–59, 60–69, 70–79, and ≥ 80 years), sex, anatomic site (ICC, ECC, GBC, and AVC), geographic region (state), and race/ethnicity (White, Hispanic, Black, American Indian/Alaska Native, and Asian/Pacific Islander), including rate ratios using White individuals as the reference group. Survival was also stratified by BTC cancer stage (localized, regional, and distant). For mortality analyses, BTC, ICC and GBC were evaluated using NPCR-SEER data while ECC, AVC and Overlapping & NOS mortality data were generated based on the SEER Incidence-based mortality database, as NPCR-SEER does not distinguish between the latter sites. In the SEER database, Hispanic and Asian individuals are more likely than White or Black individuals to be lost to follow-up; this creates inflated survival estimates for Hispanic and Asian populations [31]. To assess the impact of bias by race/ethnicity in mortality analyses, we assessed the loss to follow-up in each race/ethnicity group and calculated the adjusted survival estimates if all race/ethnicity groups had the same loss to follow-up probability (Supplementary Methods, Additional File 1). Estimates of annual percent change (APC) in BTC incidence were calculated using the weighted least-squares method. Statistics were calculated using SEER*Stat software [32] version 8.3.8.

A joinpoint regression model was fitted to depict the trend for age-adjusted BTC incidence and mortality rates [33]. The number and location of joinpoints were identified by a sequential testing procedure [34]. The Joinpoint Regression Program 4.6.0.0 was used for these analyses.

A sensitivity analysis was performed to verify the end cases of the joinpoint analysis for ECC incidence and Overlapping & NOS incidence. Because SEER*Stat does not provide the delayed-adjusted estimates for NPCR incidence data for the whole US, trends were generated from 2000 to 2017 from the SEER 21 registry [35] for the sensitivity analysis. As the populations are different from the earlier analyses, the age-adjusted incidence rates are slightly different.

## Results

### Incidence and prevalence

From 2001 to 2015 in the US, the ASR per 100,000 person-years for BTC overall was 5.04 (95% confidence interval [95% CI], 5.02–5.06) (Table 1). Incidence was higher for males versus females overall (5.31 versus 4.85), and for all anatomic sites except GBC (Table 1). GBC and AVC had the highest and lowest overall incidence rates, respectively.

**Table 1:** Incidence rates of BTC in the United States by anatomic site and sex, ASR: 2001–2015 [26]

	**BTC,** **ASR (95% CI; *N* = 174,819)**	**GBC,** **ASR (95% CI; *N* = 54,214)**	**ICC,** **ASR (95% CI; *N* = 40,967)**	**ECC,** **ASR (95% CI; *N* = 43,188)**	**AVC,** **ASR (95% CI; *N* = 26,356)**	**Overlapping & NOS,** **ASR (95% CI; *N* = 10,094)**
Combined	5.04 (5.02 to 5.06)	1.57 (1.56 to 1.58)	1.17 (1.16 to 1.19)	1.25 (1.24 to 1.26)	0.76 (0.75 to 0.77)	0.29 (0.29 to 0.30)
Male	5.31 (5.27 to 5.34)	1.14 (1.12 to 1.16)	1.33 (1.31 to 1.35)	1.55 (1.53 to 1.57)	0.97 (0.95 to 0.98)	0.33 (0.32 to 0.34)
Female	4.85 (4.82 to 4.89)	1.92 (1.90 to 1.94)	1.05 (1.04 to 1.07)	1.02 (1.01 to 1.04)	0.61 (0.60 to 0.62)	0.26 (0.25 to 0.27)

Incidence rates are age-standardized and reported as cases per 100,000 person-years

*ASR* Age-standardized rate, *AVC* Ampulla of Vater cancer, *BTC* Biliary tract cancer, *CI* Confidence interval, *ECC* Extrahepatic cholangiocarcinoma, *GBC* Gallbladder cancer, *ICC* intrahepatic cholangiocarcinoma, *NOS* Not otherwise specified

**Fig. 1:**
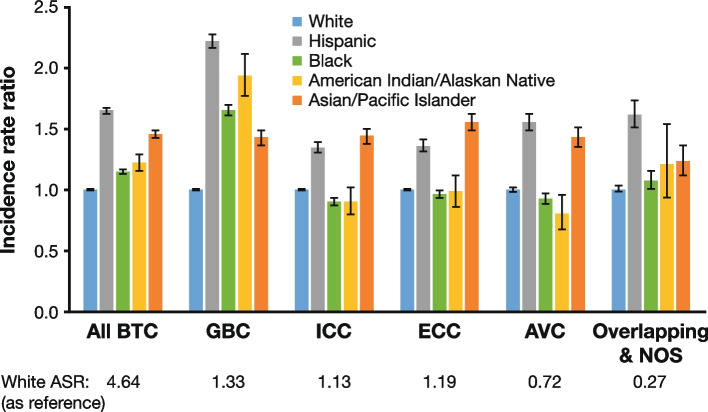
BTC incidence rate ratio of different races/ethnicities: 2001–2015 [27]. Note: Incidence rates are ASR and reported as cases per 100,000 person-years. Incidence rate ratio = ASR (race/ethnicity)/White race ASR used as a reference group. Error bars show 95% CI. *ASR* age-standardized rate, *AVC* ampulla of Vater cancer, *BTC* biliary tract cancer, *CI* confidence interval, *ECC* extrahepatic cholangiocarcinoma, *GBC* gallbladder cancer, *ICC* intrahepatic cholangiocarcinoma, *NOS* not otherwise specified

Compared with White individuals, Hispanic and Asian/Pacific Islander individuals had higher incidence across all BTC sites and were the top two race/ethnicity groups for all anatomic site categories except GBC (Fig. 1; Supplementary Table 2, Additional File 1). For GBC, Hispanic individuals had the highest incidence (2.95 [95% CI, 2.88–3.02]), followed by American Indian/Alaska Native (2.57 [95% CI, 2.36–2.80]), Black (2.19 [95% CI, 2.14–2.24]), Asian/Pacific Islander (1.89 [95% CI, 1.82–1.97]), and White (1.33 [95% CI, 1.31–1.34]) individuals (Fig. 1; Supplementary Table 2, Additional File 1).

During the analysis period, BTC total incidence increased (APC, 1.76 [95% CI, 1.59–1.92]), and the greatest change in anatomic subgroup was observed for ICC (APC, 6.65 [95% CI, 6.11–7.19]) (Supplementary Table 3, Additional File 1). APCs by sex, race/ethnicity, and age are shown in Supplementary Figs. 1–3, Additional File 1. The greatest APC in BTC by race/ethnicity was observed in Black (2.78 [95% CI, 2.23–3.34]) and White (1.62 [95% CI, 1.44–1.79]) individuals (Supplementary Fig. 2, Additional File 1). For GBC, the APC decreased in all races/ethnicities, except for Black individuals, where APC increased (1.78 [95% CI, 1.07–2.49]), and Asian/Pacific Islander individuals, where APC remained stable. GBC also decreased in individuals aged ≥ 80 years (Supplementary Fig. 3a, Additional File 1) while there is a trend toward an increasing GBC APC in individuals aged 20–59 (0.78 [95% CI, 0.38–1.18]) (Supplementary Fig. 3b, Additional File 1). ICC incidence increased regardless of age, race/ethnicity, or sex. ECC rates increased modestly overall (0.72 [95% CI, 0.34–1.10]) and for men and women, White and Black individuals, and individuals aged 50–79 years. For AVC, the incidence increased for individuals aged 40–59 years, but remained generally stable for other ages, males and females, and all races/ethnicities. The incidence of BTC diagnosed as “overlapping and biliary tract, NOS” increased across all demographic groups, except individuals aged ≥ 80 years.

**Fig. 2:**
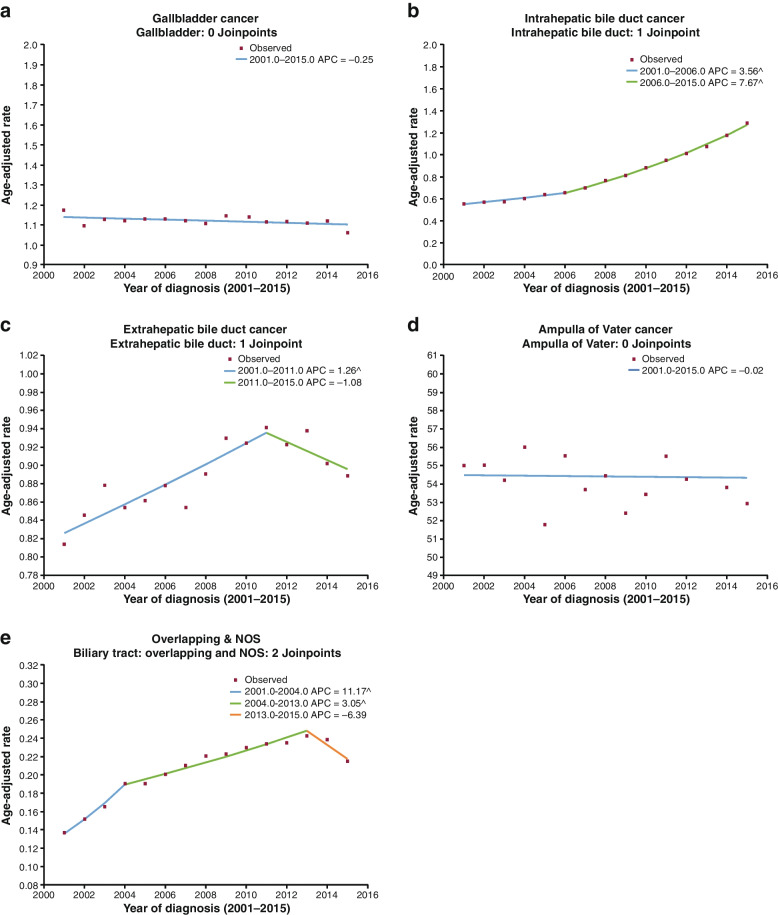
Joinpoint modeling of BTC incidence [27]. Note: ^ Indicates that the APC is significantly different from zero at the alpha = 0.05 level. *APC* annual percent change, *BTC* biliary tract cancer, *NOS* not otherwise specified

To further characterize these trends, joinpoint modeling was performed. No joinpoints were found for GBC or AVC (Fig. 2a, d). ICC incidence rose from 2001 to 2006, and then increased at a faster rate from 2006 to 2015 (Fig. 2b). ECC incidence increased from 2001 to 2011, and then decreased (Fig. 2c). For Overlapping & NOS, where the anatomic site was not indicated, incidence increased from 2001 to 2013, and then decreased (Fig. 2e). Analysis of Overlapping & NOS designation (Supplementary Fig. 4, Additional File 1) showed an increase in NOS coding.

A sensitivity analysis for ECC trend showed that instead of decreasing near the end of the period, they may be leveling off (Supplementary Fig. 5, Additional File 1), while for Overlapping & NOS, the sensitivity analysis supported the decrease in incidence from 2013 (Supplementary Fig. 6, Additional File 1).

**Fig. 3:**
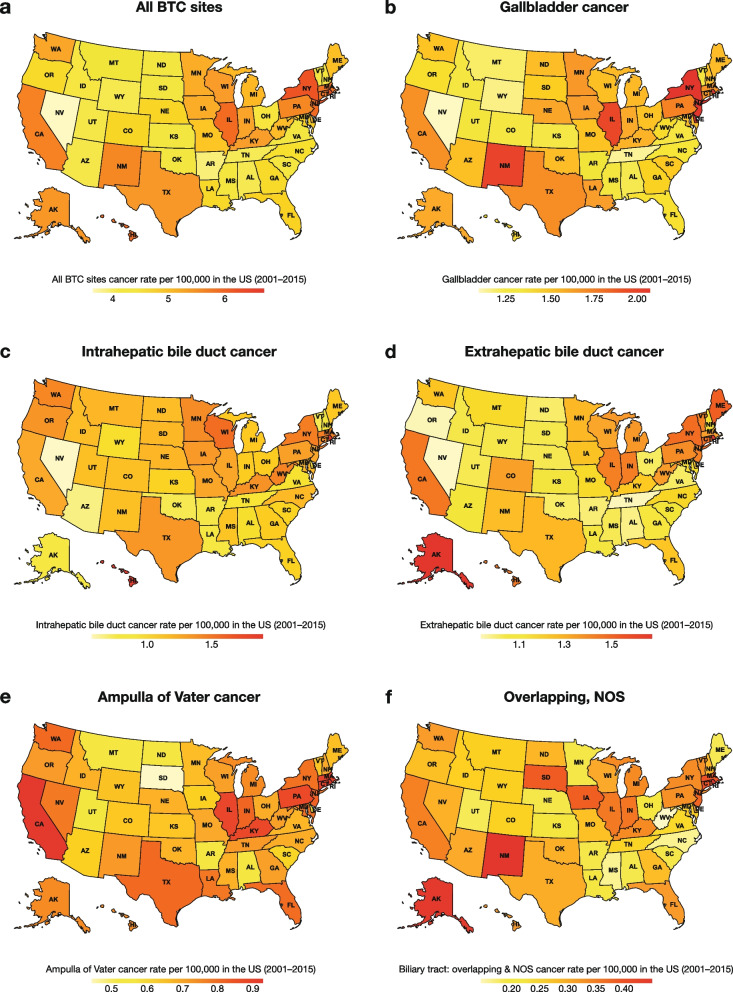
Incidence rate of BTC overall and by anatomic site in the United States: 2001–2015 [27]. Note: Incidence rates are age-standardized and reported as cases per 100,000 person-years. *BTC* biliary tract cancer, *NOS* not otherwise specified, *US* United States

BTC incidence across the US (Fig. 3a) was highest in several Northeastern states (Rhode Island, New York, New Jersey, and Massachusetts), Illinois in the Midwest, and California in the Southwest (ASR ≥ 5.5), and lowest in Nevada, Arkansas, and Tennessee (ASR < 4.0). BTC incidence by anatomic site is shown in Fig. 3b–3f. Although rates were similar, compared with BTC overall, GBC incidence was highest in New York, New Jersey, Illinois, and New Mexico (ASR > 1.9), and lowest in Nevada (ASR, 1.09). ICC incidence was highest in Rhode Island and Hawaii (ASR > 1.5), and lowest in Nevada (ASR, 0.58). ECC incidence was highest in Rhode Island and Alaska (ASR > 1.5), and lowest in Nevada, Oregon, and Tennessee (ASR ≤ 0.95). AVC incidence was highest in Rhode Island and California (ASR > 0.90), and lowest in South Dakota (ASR, 0.45).

The 10-year BTC prevalence patterns mirrored those for incidence, with an overall rate of 10.8 per 100,000 (Supplementary Table 4, Additional File 1). Hispanic and Asian/Pacific Islander individuals had higher 10-year BTC prevalence (16.6 and 13.8, respectively) than the other race/ethnicity groups (Supplementary Fig. 7; Supplementary Table 4; Additional File 1). GBC was the most prevalent type of BTC for all races/ethnicities.

### Mortality and survival

**Fig. 4:**
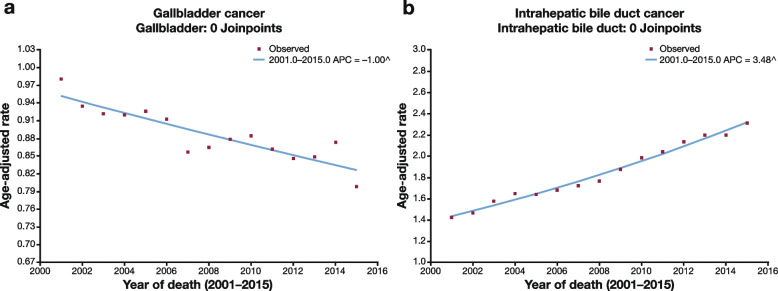
Joinpoint modeling of BTC mortality [27]. Note: ^ Indicates that the APC is significantly different from zero at the alpha = 0.05 level. *APC* annual percent change, *BTC* biliary tract cancer

The overall mortality ASR for BTC patients was 3.40 (95% CI, 3.38–3.42) (Table 2). Compared with White individuals (3.25 [95% CI, 3.23–3.28]), all other race/ethnicity groups had a higher mortality rate for BTC with the highest rates among Hispanic (4.43 [95% CI, 4.34–4.52]; rate ratio, 1.36 [95% CI, 1.33–1.39]) and Asian/Pacific Islander (4.30 [95% CI, 4.18–4.42]; rate ratio, 1.32 [95% CI, 1.28–1.36]) individuals (Table 2; Supplementary Fig. 8, Additional File 1). Among the anatomic sites, the mortality rate was highest for ICC (1.87 [95% CI, 1.85–1.88]), particularly for Asian/Pacific Islander individuals (2.57 [95% CI, 2.48–2.66]). Mortality was lowest in Overlapping & NOS (0.29 [95% CI, 0.28–0.30]), particularly for American Indian/Alaska Native individuals (0.25 [95% CI, 0.14–0.40]). Mortality decreased by 1% per year for GBC and increased by 3.5% per year for ICC without any changes observed in the rate of decrease or increase over the study period (Fig. 4).

**Table 2:** Mortality rate of BTC overall and by anatomic sites by race/ethnicity, ASR: 2001–2015 [27, 28]

A)	**ASR (95% CI)**			B)	**ASR (95% CI)**		
	**BTC** **(*N* = 117,854)**	**ICC** **(*N* = 64,882)**	**GBC** **(*N* = 30,521)**		**ECC** **(*N* = 9889)**	**AVC** **(*N* = 4774)**	**Overlapping & NOS** **(*N* = 2626)**
**All races/** **ethnicities**	3.40 (3.38 to 3.42)	1.87 (1.85 to 1.88)	0.88 (0.87 to 0.89)	**All races/** **ethnicities**	1.10 (1.08 to 1.12)	0.53 (0.52 to 0.55)	0.29 (0.28 to 0.30)
**White**	3.25 (3.23 to 3.28)	1.82 (1.80 to 1.84)	0.78 (0.77 to 0.79)	**White**	1.00 (0.98 to 1.03)	0.48 (0.47 to 0.50)	0.27 (0.26 to 0.28)
**Hispanic**	4.43 (4.34 to 4.52)	2.26 (2.20 to 2.33)	1.47 (1.42 to 1.52)	**Hispanic**	1.46 (1.38 to 1.55)	0.80 (0.74 to 0.87)	0.42 (0.38 to 0.47)
**Black**	3.48 (3.41 to 3.55)	1.71 (1.66 to 1.75)	1.21 (1.18 to 1.25)	**Black**	0.99 (0.92 to 1.06)	0.51 (0.46 to 0.56)	0.28 (0.24 to 0.32)
**American Indian/Alaska Native**	3.97 (3.69 to 4.25)	1.93 (1.74 to 2.13)	1.45 (1.28 to 1.63)	**American Indian/Alaska Native**	1.19 (0.94 to 1.48)	0.48 (0.32 to 0.67)	0.25 (0.14 to 0.40)
**Asian/Pacific Islander**	4.30 (4.18 to 4.42)	2.57 (2.48 to 2.66)	1.02 (0.96 to 1.08)	**Asian/Pacific Islander**	1.60 (1.51 to 1.69)	0.64 (0.58 to 0.70)	0.31 (0.27 to 0.35)

Mortality rates are age-standardized and reported as deaths per 100,000 person-years. A) Mortality rates for total BTC, ICC and GBC, evaluated using NPCR-SEER data. Total BTC rates from the NPCR-SEER database includes ICC, GBC, and Other Biliary Cancers. B) ECC, AVC and Overlapping & NOS mortality data, evaluated using the SEER 18 Incidence-based mortality database

*ASR* Age-standardized rate, *BTC* Biliary tract cancer, *CI* Confidence interval, *GBC* Gallbladder cancer, *ICC* Intrahepatic cholangiocarcinoma, *SEER* Surveillance, Epidemiology, and End Results

BTC mortality across the US (Supplementary Fig. 9) was highest in several Northeastern (Rhode Island, Massachusetts), Midwestern (Minnesota, Wisconsin, Illinois), and Western (New Mexico, Hawaii, Washington) states (ASR > 3.8). BTC mortality was lowest in several Southern (Mississippi, Arkansas, Alabama, Louisiana, Oklahoma) and Western (Nevada, Utah, Arizona) states (ASR < 2.8). Rates of BTC mortality by anatomic site varied compared with BTC overall. GBC mortality was highest in New York, Illinois, the Northeastern State of New Jersey, Minnesota, New Mexico, and the Southern state of Delaware (ASR > 1.0), and lowest in Utah and Wyoming in the West (ASR < 0.6). ICC mortality was highest in Hawaii, Rhode Island, Washington, Connecticut, Wisconsin, and Minnesota (ASR > 2.3) and lowest in Arkansas and Mississippi (ASR < 1.1). In Other Biliary Cancers, mortality rates were highest in Rhode Island, the Northeastern state of Vermont, New Mexico and the Midwestern state of Nebraska (ASR > 0.8) and generally low in most states, with the lowest rate in Alabama (ASR = 0.52).

**Fig. 5:**
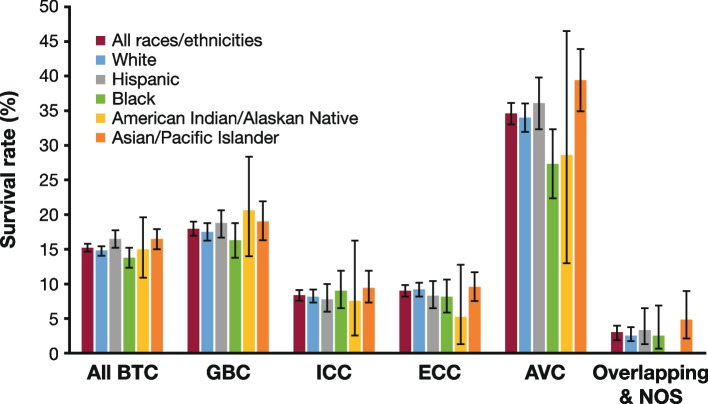
Five-year relative survival for patients with BTC by anatomic site and race/ethnicity [29]. Note: Error bars show 95% CI. *AVC* ampulla of Vater cancer, *BTC* biliary tract cancer, *CI* confidence interval, *ECC* extrahepatic cholangiocarcinoma, *GBC* gallbladder cancer, *ICC* intrahepatic cholangiocarcinoma, *NOS* not otherwise specified

Five-year survival for BTC was 15.2% (95% CI, 14.8–15.7). The lowest survival was reported for ICC (8.5% [95% CI, 7.8–9.2]) and the highest for AVC (34.5% [95% CI, 33.0–36.0]) (Fig. 5; Supplementary Table 5, Additional File 1). BTC survival among race/ethnicity groups were largely consistent with the overall population across anatomic site. Black individuals had the lowest survival (13.8% [95% CI, 12.4–15.2]), and Hispanic individuals had the highest (16.6% [95% CI, 15.4–17.7]) (Fig. 5, Supplementary Table 5). Loss to follow-up varied by race/ethnicity and stage (Additional File 1, Supplementary Table 6). After adjusting for loss to follow-up, all 5-year relative survival estimates were lower than the original estimates (Supplementary Table 7). Black individuals still had the lowest survival (12.5%), but Hispanics no longer had the highest survival. Instead, American Indian/Alaska Native individuals had the highest survival (14.7%), demonstrating how loss to follow-up can bias survival estimates (Additional File 1, Supplementary Table 7). Five-year survival was low for patients with distant disease at first diagnosis (3.0% [95% CI, 2.6–3.4]), compared with regional (19.1% [95% CI, 18.3–20.0]) or localized (31.5% [95% CI, 30.3–32.7]) disease (Supplementary Fig. 10, Additional File 1).

## Discussion

This study reports the incidence, prevalence, mortality, and survival for BTC overall and for each anatomic site using cancer registry data that are representative of the US population.

BTC incidence increased from 2001 to 2015; the highest rates were observed in Hispanic and Asian/Pacific Islander individuals, while the greatest increase in incidence over time was among Black individuals. BTC mortality was highest in Hispanic and Asian/Pacific Islander individuals. Five-year survival for BTC was poor, with a marked difference for patients with distant disease at diagnosis compared with regional or localized disease (3.0% versus 19.1% and 31.5%). After adjusting for loss to follow-up, Black individuals also had the poorest five-year survival. Our findings are consistent with other studies showing an increase in BTC incidence and low survival [4, 36–38].

GBC incidence and prevalence were the highest of the BTC subtypes. GBC incidence and prevalence were higher in Hispanic individuals, which is consistent with the higher rate of GBC observed in several Latin American countries [39]. In our study, GBC incidence decreased or remained stable, except in Black individuals, among whom it increased between 2001 and 2015. This finding is consistent with a report that used registry data from 38 states and examined BTC incidence from 1999 to 2013 [4]. They reported GBC incidence in Black individuals increased by 1.8% per year during the study period, although the incidence of GBC declined overall by 0.4% per year, and among White, Hispanic, and other races/ethnicities by 0.9%, 1.8%, and 1.2% per year, respectively [4]. The overall decrease in GBC incidence may be driven by decreasing incidence in older age groups, where there are more cases. We observed a 1% per year decrease in GBC mortality, which was consistent with the decreasing GBC mortality rate observed in most countries by L. A. Torre et al. [25].

ICC incidence increased the most of all subtypes — 6.7% per year, from 2001 to 2015. Similarly, A. L. Van Dyke et al. found that ICC increased by 3.2% per year from 1999 to 2013 [4]. The higher rate in our analysis may be due to differences in the included states. Additionally, our joinpoint analysis showed an acceleration of the increase in ICC rate after 2006, likely due to inclusion of data up to 2015. Mortality rate and 5-year survival for ICC were poorest among the BTC subtypes in our analysis.

While prevalence of ECC was the lowest among all BTC, it increased by 0.72% per year from 2001 to 2015. Similarly, A. L. Van Dyke et al. found that ECC increased by 1.8% per year from 1999 to 2013 [4]. However, conflicting studies have reported that ECC incidence and mortality rates were stable or decreasing [26, 40]. S. K. Saha et al. found ECC incidence was stable from 1973 to 2012, using data from the SEER database, although the coding approach in this study differed from that presented here [40]. The incidence rate ratio trends for ECC by race/ethnicity showed that, compared with White individuals, the incidence in Black individuals increased over time [40], consistent with our findings. Additionally, our joinpoint and sensitivity analyses showed that ECC incidence leveled out after 2003, consistent with the results presented by S. K. Saha et al. [40]. Five-year survival for ECC was the second lowest and approached that of ICC. Unfortunately, ECC mortality was not assessed by anatomic site and is not defined in cause of death in the SEER mortality database. However, P. Bertuccio et al. demonstrated that, on a global scale, mortality from ECC decreased in most countries from 1995 to 2016 [26].

Incidence rate was lowest for AVC, highlighting its rarity. Five-year survival of AVC was highest among BTC subtypes and exceeded the < 20% 5-year survival observed in other subtypes. Similarly to ECC, assessment of AVC-specific mortality was prevented due to anatomic site not being defined in cause of death in the SEER database.

Our data are in agreement with, and build upon, that presented by T. D. Ellington et al. [41] in a study of BTC incidence in the US between 2013–2017 using population-based data from the NPCR-SEER dataset. Age-adjusted incidence of AVC was rare, at 0.45 per 100,000 standard population whilst ICC, GBC and ECC were more common (ASRs of 1.49, 1.11 and 0.96 per 100,000 standard population, respectively). Similar to our findings, incidence was also higher in males versus females and Hispanic or Asian/Pacific Islander individuals versus all races/ethnicities combined for all anatomic sites except GBC. However, unlike T. D. Ellington et al., our study also provided joinpoint analysis of incidence and mortality over time; an important addition given the differences observed in temporal trend by anatomic subgroup and subpopulation.

Interestingly, we found that the incidence of Overlapping & NOS lesions increased over time, due to an increase in NOS, not overlapping lesions. However, joinpoint and sensitivity analyses showed that since 2012 the use of NOS may be decreasing. This decreasing trend coincides with the start of next generation sequencing and recognition of the molecular landscape in BTC, which have been instrumental in the drive to specify anatomic subsite. Similarly, the number of clinical trials increased dramatically around 2010, many of which require subsite specifications. Accurate site specification of BTC continues to be important for diagnosis and treatment and will benefit from a continued reduction in NOS coding.

Many factors may impact the incidence, prevalence, mortality, and survival trends of BTC observed in this study. Increasing rates of obesity and fatty liver disease, which have been associated with increased risk of BTC, may contribute to rising disease incidence [42–44]. As we found a higher incidence of BTC in Hispanic, Asian/Pacific Islander, Black and American Indian/Alaska Native individuals, compared with White individuals, population differences may further account for the geographic differences in incidence observed. In particular, there is a greater Hispanic population in California and Native American population in New Mexico, two states with high BTC incidence rates [3, 7]. Discrepancies among race/ethnicity in the US may reflect socioeconomic and healthcare access differences or may reflect improved reporting or diagnosis over time. One study showed race/ethnicity and socioeconomic disparities in the receipt of initial treatment of cholangiocarcinoma, but not for receipt of multimodality therapy (radiation in patients who received chemotherapy and radiation and/or chemotherapy in post-surgical patients) [45]. These data suggest a potential barrier to initial access to care or navigation of the healthcare system for these patients; once patients accessed the healthcare system, all races/ethnicities appeared to receive treatment at the same rate. The observed geographic differences in incidence support that differences in exposure, population, socioeconomic status, and access to care between states may account for some of the observed variation.

While hypotheses can be made, the descriptive design of this study prevents direct evaluation of explanations for the observed trends and patterns of BTC incidence, prevalence, mortality, and survival. Prevalence and survival data are taken from the SEER 18 database, representing a subset of the US population, and thus results may not be generalizable to the entire population. Another caveat of using the NPCR database is that the completeness of data varies by state; for example, Nevada was only certified by NPCR in 2017, limiting the accuracy of comparisons by state. However, removal of a single state with a small population, such as Nevada, will not significantly impact national incidence. Thus, all states were included in the analysis. Additionally, mortality data for all BTC, ICC and GBC were taken from the NPCR-SEER database, while mortality data for ECC, AVC, and Overlapping & NOS were generated based on the SEER Incidence-based mortality database, meaning rates between subtypes may not be directly comparable. Another limitation of our study is that the denominator in the analysis may include individuals who have had a cholecystectomy and who are not at risk of developing GBC because their gallbladder has been removed. Finally, Hispanic and Asian/Pacific Islander individuals are more likely to be lost to follow-up compared with White or Black individuals and may be falsely assumed to be surviving [31]. This may result in biases that produce an overestimation of survival among Hispanic and Asian/Pacific Islander individuals [31]. While we addressed this bias by calculating adjusted survival estimates based on loss to follow-up probability, the survival rates reported for Hispanic and Asian/Pacific Islander individuals are likely to be overestimated and should be interpreted with caution.

Molecular characteristics of BTC vary by geographic and ethnic populations, as demonstrated in ICC: tumors from Chinese individuals have been found to have a higher rate of DNA repair gene mutations and a higher tumor mutational burden, compared with a mostly White population located in the US [22]. In contrast, alterations in actionable driver genes were more common in patients in the US. These differences may be due to differing etiologies in Asia (liver fluke, hepatitis B virus), compared with Western populations (metabolic syndrome, inflammatory bowel disease) [22]. Further molecular studies on sub-populations within the US are needed to better understand differences in molecular characteristics by ethnicity. Evaluation of tumors arising in populations with increasing incidence rates may also identify unique traits that are amenable to targeted treatments. Furthermore, as genetic features vary by anatomic site in the biliary tract, it is critical to accurately identify the anatomic origin of the tumor.

## Conclusions

From 2001 to 2015 in the US, BTC incidence increased, with the greatest increase occurring in ICC. Hispanic and Asian/Pacific Islanders generally had the highest incidence rates, while the greatest APC was found in Black individuals. Survival was low across all subtypes and races/ethnicities, with lowest survival observed amongst those with distant disease at diagnosis. The US BTC incidence and mortality patterns reported here underscore the serious and unmet need among patients with BTC overall, and for each subtype. With limited treatment options for BTC, insights into its epidemiology will inform healthcare providers to help patients access better care and enable the integration of novel therapies.

## Supplementary Information

**Additional file 1: Supplementary Methods. Supplementary Table 1.** Codes used for cancer classifications. **Supplementary Table 2.** BTC incidence rate by race/ethnicity: 2001–2015. **Supplementary Table 3.** Annual percent change in BTC rates: 2001–2015. **Supplementary Table 4.** The 10-year prevalence of BTC overall and by anatomic site across different races/ethnicities in 2015. **Supplementary Table 5.** Five-year relative survival for patients with BTC overall and by anatomic site across different races/ethnicities. **Supplementary Table 6.** Percentage lost to follow-up by race/ethnicity and historic stage. **Supplementary Table 7.** Original and adjusted 5-year relative survival (%) after adjusting for higher lost-to-follow-up for the race-ethnicity minority groups. **Supplementary Fig. 1. **Comparison of APC by sex. **Supplementary Fig. 2.** Comparison of APC by race/ethnicity. **Supplementary Fig. 3.** Comparison of APC by age group. **Supplementary Fig. 4. **BTC incidence: overlapping & NOS. **Supplementary Fig. 5. **Sensitivity analysis of extrahepatic bile duct incidence. **Supplementary Fig. 6. **Sensitivity analysis of overlapping & NOS incidence. **Supplementary Fig. 7. **The 10-year prevalence of BTC by anatomic site and race/ethnicity in 2015. **Supplementary Fig. 8.** Mortality rate trends. **Supplementary Fig 9.** Mortality rate of BTC overall and by anatomic site in the United States: 2001–2015. **Supplementary Fig. 10.** Comparisons of relative survival between historical stages.

**Additional file 2. **Original survival data for the Asian/Pacific Islander population with distant BTC and the SEER*Stat Survival Session.

## Data Availability

The data that support the findings of this study are available from the NPCR-SEER Program of the National Cancer Institute (NCI), but restrictions apply to the availability of these data, which were used under license for the current study. Data for this analysis are however available from the authors upon reasonable request and with permission of the NCI*.* Please contact the corresponding author for further information.

## Abbreviations

APC: Annual percent change
ASR: Age-standardized rate
AVC: Ampulla of Vater cancer
BTC: Biliary tract cancer
CI: Confidence interval
ECC: Extrahepatic cholangiocarcinoma
FGFR: Fibroblast growth factor receptor
GBC: Gallbladder cancer
ICC: Intrahepatic cholangiocarcinoma
IDH1: Isocitrate dehydrogenase 1
mOS: Median overall survival
mPFS: Median progression-free survival
NOS: Not otherwise specified
NPCR: National Program of Cancer Registries
SEER: Surveillance, Epidemiology, and End Results
