# Supplementary material for: Epidemiologic patterns of biliary tract cancer in the United States: 2001–2015
Source: BMC Cancer. 2022 Nov 16;22:1178. doi: 10.1186/s12885-022-10286-z (PMC9670575; doi:10.1186/s12885-022-10286-z)
Supplement: Supplementary file 1 — Additional file 1: Supplementary Methods. Supplementary Table 1. Codes used for cancer classifications. Supplementary Table 2. BTC incidence rate by race/ethnicity: 2001–2015. Supplementary Table 3. Annual percent change in BTC rates: 2001–2015. Supplementary Table 4. The 10-year prevalence of BTC overall and by anatomic site across different races/ethnicities in 2015. Supplementary Table 5. Five-year relative survival for patients with BTC overall and by anatomic site across different races/ethnicities. Supplementary Table 6. Percentage lost to follow-up by race/ethnicity and historic stage. Supplementary Table 7. Original and adjusted 5-year relative survival (%) after adjusting for higher lost-to-follow-up for the race-ethnicity minority groups. Supplementary Fig. 1. Comparison of APC by sex. Supplementary Fig. 2. Comparison of APC by race/ethnicity. Supplementary Fig. 3. Comparison of APC by age group. Supplementary Fig. 4. BTC incidence: overlapping & NOS. Supplementary Fig. 5. Sensitivity analysis of extrahepatic bile duct incidence. Supplementary Fig. 6. Sensitivity analysis of overlapping & NOS incidence. Supplementary Fig. 7. The 10-year prevalence of BTC by anatomic site and race/ethnicity in 2015. Supplementary Fig. 8. Mortality rate trends. Supplementary Fig 9. Mortality rate of BTC overall and by anatomic site in the United States: 2001–2015. Supplementary Fig. 10. Comparisons of relative survival between historical stages. [file 12885_2022_10286_MOESM1_ESM.docx]

**Additional File 1**

**Supplementary Methods**

**Data Sources**

The National Program of Cancer Registries (NPCR), which is administered by the Centers for Disease Control and Prevention, collects data on cancer occurrence (including the type, extent, and location of the cancer), type of initial treatment, and outcomes in the United States [1]. The complementary Surveillance, Epidemiology, and End Results (SEER) Program, supported by the National Cancer Institute, provides information on cancer statistics for the US population. Combined data from the NPCR and SEER Program provide cancer incidence and mortality data for all 50 states, the District of Columbia, and Puerto Rico, representing over 22 million cancer cases. For the current study, the incidence and mortality data of BTC were obtained from the 2001–2015 NPCR-SEER Database [2], however, the NPCR-SEER mortality database does not separate by all anatomic sites (extrahepatic cholangiocarcinoma [ECC], Ampulla of Vater cancer [AVC] and any overlapping or not otherwise specified cancers [Overlapping & NOS] are grouped under ‘Other Biliary Cancers’) thus, mortality data for ECC, AVC and Overlapping & NOS were taken from the SEER 18 Incidence-based mortality database [3] . Prevalence and survival data were obtained from the 2001–2015 SEER 18 database [4, 5]. Institutional Review Board approval was not needed due to the use of secondary deidentified data.

**Cancer Classifications**

Eligible patients were aged ≥20 years (pediatric patients are rare [6] and SEER reports the data for adults aged 18 and 19 years with a subset of pediatric patients) and diagnosed with BTC as their primary cancer (malignant tumor) according to the codes below. Patients with incident BTC were identified using the International Classification of Diseases for Oncology, Third Edition (ICD-O-3) codes and morphology codes [7], as follows: ECC (topography code C24.0 plus morphology code 8032/3, 8070/1, 8140, 8160, 8162/3, 8260, 8480, 8481, 8490, or 8560), ICC (topography code C22.1 plus morphology code 8032/3, 8070/1, 8140, 8160, 8260, 8480, 8481, 8490, or 8560; topography code C22.0 plus morphology code 8160/3), GBC (C23.9), AVC (C24.1), and overlapping and biliary tract, not otherwise specified (NOS), where the anatomical location is not identified (C24.8 and C24.9). Klatskin tumors with morphology code 8162/3, and any topography code, were included with ECC. Overlapping and NOS were combined into a single category for analysis. Lymphohematologic tumors [8] were excluded (Supplementary Table 1); this equates to 660 patients. These codes were used for the incidence, prevalence, and survival. Mortality due to BTC was identified through ICD-9 [9] and ICD-10 [10] codes for the causes of death listed in Supplementary Table 1. Patients were excluded if diagnosis was post-mortem (approximately 2% of cancer cases in the NPCR-SEER database, diagnosed from 2002–2006, were diagnosed by death certificate or post-mortem only[11]) or diagnosis year was unknown, however, there were no missing values in diagnosis year.

**Impact of loss to follow-up on the survival estimates for Hispanic and Asian/Pacific Islander individuals**

The population-based survival data are grouped into intervals $I_{j}=(t_{j-1},t_{j}),j=1,...,J$ where $t_{0}=0$ and $t_{J}=B$. The actuarial estimate of overall survival is

$$S(t_{j})=\prod_{k=1,...,j} (1-\frac{d_{k}}{n_{k}-\frac{1}{2}r_{k}})=\prod_{k=1,...,j} (1-\frac{d_{k}}{n_{k}-c_{k}}),(1)$$

where $n_{k}$ is the number of subjects alive at the beginning of interval $I_{k}$, and $r_{k}$ and $d_{k}$ are the numbers of subjects who are lost to follow-up or die during interval $I_{k}$. The adjusted number of censoring is $c_{k}=\frac{1}{2}r_{k}$. If some factors, for example race/ethnicity, are associated with both death and censoring times, as has been suggested for cancer survival [12], the Kaplan-Meier and actuarial survival estimators, which assume independent censoring, are biased. Particularly, the Kaplan-Meier estimate over-estimates the real survival estimate [13]. The actuarial estimate has the same problem. We thus asked, if all race/ethnicity groups had the same lost to-follow up probability as White individuals, the largest race/ethnicity group, what would be the adjusted survival estimates for the race/ethnicity minority groups?

Let $Z=0$ denote the control group, White individuals, and $Z=1$ denote the other race/ethnicity to be adjusted. For group $Z=z (z=0,1)$, $n_{zj},d_{zj},r_{zj}$ are the number of subjects alive at the beginning of, dying during and lost to follow-up during interval $I_{j}$, respectively. The sensitivity analysis is conducted as follows:

1. For $Z=0$ (White), calculate the probability of lost to follow-up during interval $I_{j}$ for subjects when they are at risk in the beginning of interval $I_{j}$: $P_{0j}=r_{0j}/n_{0j}$. For $Z=1$ (race/ethnicity minority), $P_{1j}=r_{1j}/n_{1j}$, calculate the inverse probability censoring/lost-to-follow-up weight (IPCW) as $W_{j}=\frac{P_{0j}}{P_{1j}}$.
2. After adjusting for the differential probabilities of lost to follow-up, the expected number of lost to follow-up for $Z=1$ (race/ethnicity minority) is $r_{1j}^{*}=r_{1j}W_{j}$and the adjusted number of deaths $d_{zj}^{*}=max(0,r_{zj}+d_{zj}-r_{zj}^{*})$
3. Estimate corrected survival, where $S(t_{j}|Z=1)=\prod_{k=1,...,j} (1-\frac{d_{1k}^{*}}{n_{1k}-\frac{1}{2}r_{1k}^{*}})$

**Supplementary Tables**

**Supplementary Table 1** Codes used for cancer classifications

| Mortality | | | | | |
| --- | --- | --- | --- | --- | --- |
| Cancer causes of death | ICD-9 (1979–1988) codes [6] | | ICD-10 (1999+) codes [7] | | Recode |
| Liver | 155.0, 155.2 | | C22.0, C22.2-C22.4, C22.7, C22.9 | | 21071 |
| Intrahepatic Bile Duct | 155.1 | | C22.1 | | 21072 |
| Gallbladder | 156.0 | | C23 | | 21080 |
| Other Biliary | 156.1-156.2, 156.8-156.9 | | C24 | | 21090 |
| List of excluded lymphohematologic tumors (3) | | | | | |
| ICD-O-3 code | | Name | | Reportability | |
| 9737/3 | | ALK-positive large B-cell lymphoma | | 2010 and later | |
| 9870/3 | | Acute basophilic leukemia | | 1978 and later | |
| 9805/3 | | Acute biphenotypic leukemia | | 2001-2009 | |
| 9910/3 | | Acute megakaryoblastic leukemia | | 1978 and later | |
| 9891/3 | | Acute monoblastic and monocytic leukemia | | 1978 and later | |
| 9911/3 | | Acute myeloid leukemia (megakaryoblastic) with t(1;22)(p13.3;q13.1); RBM15-MKL1 | | 2010 and later | |
| 9912/3 | | Acute myeloid leukemia with BCR-ABL1 | | 2021 and later | |
| 9878/3 | | Acute myeloid leukemia with biallelic mutation of CEBPA | | 2021 and later | |
| 9871/3 | | Acute myeloid leukemia with inv(16)(p13.1q22) or t(16;16)(p13.1;q22); CBFB-MYH11 | | 2001 and later | |
| 9869/3 | | Acute myeloid leukemia with inv(3)(q21.3q26.2) or t(3;3)(q21.3;q26.2); GATA2, MECOM | | 2010 and later | |
| 9874/3 | | Acute myeloid leukemia with maturation | | 1995 and later | |
| 9872/3 | | Acute myeloid leukemia with minimal differentiation | | 1995 and later | |
| 9877/3 | | Acute myeloid leukemia with mutated NPM1 | | 2021 and later | |
| 9879/3 | | Acute myeloid leukemia with mutated RUNX1 | | 2021 and later | |
| 9895/3 | | Acute myeloid leukemia with myelodysplasia-related changes | | 2001 and later | |
| 9865/3 | | Acute myeloid leukemia with t(6;9)(p23;q34.1); DEK-NUP214 | | 2010 and later | |
| 9897/3 | | Acute myeloid leukemia with t(9;11)(p21.3;q23.3); KMT2A-MLLT3 | | 1978 and later | |
| 9873/3 | | Acute myeloid leukemia without maturation | | 2001 and later | |
| 9861/3 | | Acute myeloid leukemia, NOS | | 1978 and later | |
| 9896/3 | | Acute myeloid leukemia, t(8;21)(q22;q22.1); RUNX1-RUNX1T1 | | 2001 and later | |
| 9867/3 | | Acute myelomonocytic leukemia | | 1978 and later | |
| 9931/3 | | Acute panmyelosis with myelofibrosis | | 1992 and later | |
| 9866/3 | | Acute promyelocytic leukemia with PML-RARA | | 1978 and later | |
| 9801/3 | | Acute undifferentiated leukemia | | 1978 and later | |
| 9827/3 | | Adult T-cell leukemia/lymphoma | | 1992 and later | |
| 9948/3 | | Aggressive NK-cell leukemia | | 2001 and later | |
| 9715/3 | | Anaplastic large cell lymphoma, ALK-negative | | 2021 and later | |
| 9714/3 | | Anaplastic large cell lymphoma, ALK-positive | | 1992 and later | |
| 9705/3 | | Angioimmunoblastic T-cell lymphoma | | 1992 and later | |
| 9767/1 | | Angioimmunoblastic lymphadenopathy | | This neoplasm is not reportable | |
| 9876/3 | | Atypical chronic myeloid leukemia, BCR-ABL1-negative | | 2001 and later | |
| 9596/3 | | B-cell lymphoma, unclassifiable, with features intermediate between diffuse large B-cell lymphoma and classic Hodgkin lymphoma | | 2001 and later | |
| 9833/3 | | B-cell prolymphocytic leukemia | | 2001 and later | |
| 9815/3 | | B-lymphoblastic leukemia/lymphoma with hyperdiploidy | | 2010 and later | |
| 9816/3 | | B-lymphoblastic leukemia/lymphoma with hypodiploidy | | 2010 and later | |
| 9814/3 | | B-lymphoblastic leukemia/lymphoma with t(12;21) (p13.2;q22.1); ETV6-RUNX1 | | 2010 and later | |
| 9818/3 | | B-lymphoblastic leukemia/lymphoma with t(1;19)(q23;p13.3); TCF3-PBX1 | | 2010 and later | |
| 9817/3 | | B-lymphoblastic leukemia/lymphoma with t(5;14)(q31.1;q32.1); IGH/IL3 | | 2010 and later | |
| 9812/3 | | B-lymphoblastic leukemia/lymphoma with t(9;22)(q34.1;q11.2); BCR-ABL1 | | 2010 and later | |
| 9813/3 | | B-lymphoblastic leukemia/lymphoma with t(v;11q23.3); KMT2A-rearranged | | 2010 and later | |
| 9819/3 | | B-lymphoblastic leukemia/lymphoma, BCR-ABL1-like | | 2021 and later | |
| 9811/3 | | B-lymphoblastic leukemia/lymphoma, NOS | | 2010 and later | |
| 9727/3 | | Blastic plasmacytoid dendritic cell neoplasm | | 2001 and later | |
| 9826/3 | | Burkitt cell leukemia | | 1992-2020 | |
| 9687/3 | | Burkitt lymphoma | | 1978 and later | |
| 9964/3 | | Chronic eosinophilic leukemia, NOS | | 1978 and later | |
| 9823/3 | | Chronic lymphocytic leukemia/small lymphocytic lymphoma | | 1978 and later | |
| 9875/3 | | Chronic myeloid leukemia, BCR-ABL1-positive | | 2001 and later | |
| 9863/3 | | Chronic myeloid leukemia, NOS | | 1978 and later | |
| 9945/3 | | Chronic myelomonocytic leukemia | | 1992 and later | |
| 9963/3 | | Chronic neutrophilic leukemia | | 1978 and later | |
| 9650/3 | | Classic Hodgkin lymphoma | | 1978 and later | |
| 9740/1 | | Cutaneous mastocytosis | | This neoplasm is not reportable | |
| 9680/3 | | Diffuse large B-cell lymphoma, NOS | | 1992 and later | |
| 9680/1 | | EBV-positive mucocutaneous ulcer | | This neoplasm is not reportable | |
| 9717/3 | | Enteropathy-associated T-cell lymphoma | | 2001 and later | |
| 9749/3 | | Erdheim-Chester disease | | 2021 and later | |
| 9962/3 | | Essential thrombocythemia | | 2001 and later | |
| 9719/3 | | Extranodal NK-/T-cell lymphoma, nasal type | | 2001 and later | |
| 9699/3 | | Extranodal marginal zone lymphoma of mucosa-associated lymphoid tissue (MALT lymphoma) | | 1992 and later | |
| 9734/3 | | Extraosseous plasmacytoma | | 1978 and later | |
| 9759/3 | | Fibroblastic reticular cell tumor | | 2010 and later | |
| 9758/3 | | Follicular dendritic cell sarcoma | | 2001 and later | |
| 9690/3 | | Follicular lymphoma | | 1992 and later | |
| 9695/3 | | Follicular lymphoma, grade 1 | | 1978 and later | |
| 9691/3 | | Follicular lymphoma, grade 2 | | 1992 and later | |
| 9698/3 | | Follicular lymphoma, grade 3 | | 1978 and later | |
| 9738/3 | | HHV8-positive diffuse large B-cell lymphoma, NOS | | 2010 and later | |
| 9738/1 | | HHV8-positive germinotropic lymphoproliferative disorder | | This neoplasm is not reportable | |
| 9940/3 | | Hairy cell leukemia | | 1978 and later | |
| 9762/3 | | Heavy chain diseases | | 1992 and later | |
| 9716/3 | | Hepatosplenic T-cell lymphoma | | 2001 and later | |
| 9755/3 | | Histiocytic sarcoma | | 1992 and later | |
| 9661/3 | | Hodgkin granuloma | | 2001-2009 | |
| 9654/3 | | Hodgkin lymphoma, lymphocyte depletion, diffuse fibrosis | | 1978-2009 | |
| 9655/3 | | Hodgkin lymphoma, lymphocyte depletion, reticular | | 1978 and later | |
| 9664/3 | | Hodgkin lymphoma, nodular sclerosis, cellular phase | | 1978-2009 | |
| 9665/3 | | Hodgkin lymphoma, nodular sclerosis, grade 1 | | 1992-2009 | |
| 9667/3 | | Hodgkin lymphoma, nodular sclerosis, grade 2 | | 1978-2009 | |
| 9662/3 | | Hodgkin sarcoma | | 2001-2009 | |
| 9725/1 | | Hydroa vacciniforme-like lymphoproliferative disorder | | This neoplasm is not reportable | |
| 9725/3 | | Hydroa vacciniforme-like lymphoma | | 2010-2020 | |
| 9761/1 | | IgM monoclonal gammopathy of undetermined significance | | This neoplasm is not reportable | |
| 9760/3 | | Immunoproliferative disease, NOS | | 1992-2009 | |
| 9764/3 | | Immunoproliferative small intestinal disease | | 1992-2009 | |
| 9695/1 | | In situ follicular neoplasia | | This neoplasm is not reportable | |
| 9673/1 | | In situ mantle cell neoplasia | | This neoplasm is not reportable | |
| 9757/3 | | Indeterminate dendritic cell tumor | | 2001 and later | |
| 9702/1 | | Indolent T-cell lymphoproliferative disorder of the gastrointestinal tract | | This neoplasm is not reportable | |
| 9741/1 | | Indolent systemic mastocytosis | | This neoplasm is not reportable | |
| 9712/3 | | Intravascular large B-cell lymphoma | | 2010 and later | |
| 9946/3 | | Juvenile myelomonocytic leukemia | | 2001 and later | |
| 9751/1 | | LCH, NOS | | This neoplasm is not reportable | |
| 9751/3 | | LCH, disseminated | | 1978-2000, 2010 and later | |
| 9754/3 | | Langerhans cell histiocytosis, disseminated | | 1978-2009 | |
| 9753/1 | | Langerhans cell histiocytosis, multifocal | | This neoplasm is not reportable | |
| 9752/1 | | Langerhans cell histiocytosis, unifocal | | This neoplasm is not reportable | |
| 9756/3 | | Langerhans cell sarcoma | | 2001 and later | |
| 9800/3 | | Leukemia, NOS | | 1978 and later | |
| 9653/3 | | Lymphocyte-depleted classic Hodgkin lymphoma | | 1978 and later | |
| 9651/3 | | Lymphocyte-rich classic Hodgkin lymphoma | | 1992 and later | |
| 9820/3 | | Lymphoid leukemia, NOS | | 1978 and later | |
| 9766/3 | | Lymphomatoid granulomatosis grade 3 | | 2021 and later | |
| 9766/1 | | Lymphomatoid granulomatosis, NOS | | This neoplasm is not reportable | |
| 9718/1 | | Lymphomatoid papulosis | | This neoplasm is not reportable | |
| 9671/3 | | Lymphoplasmacytic lymphoma | | 1978 and later | |
| 9970/1 | | Lymphoproliferative disorder, NOS | | This neoplasm is not reportable | |
| 9750/3 | | Malignant histiocytosis | | 1978-2009 | |
| 9590/3 | | Malignant lymphoma, NOS | | 1978 and later | |
| 9684/3 | | Malignant lymphoma, large B-cell, diffuse, immunoblastic, NOS | | 1992-2009 | |
| 9675/3 | | Malignant lymphoma, mixed small and large cell, diffuse | | 1992-2009 | |
| 9670/3 | | Malignant lymphoma, small B lymphocytic, NOS | | 1978-2009 | |
| 9673/3 | | Mantle cell lymphoma | | 1992 and later | |
| 9742/3 | | Mast cell leukemia | | 1978 and later | |
| 9740/3 | | Mast cell sarcoma | | 1978 and later | |
| 9652/3 | | Mixed cellularity classic Hodgkin lymphoma | | 1978 and later | |
| 9806/3 | | Mixed-phenotype acute leukemia with t(9;22)(q34.1;q11.2); BCR-ABL1 | | 2010 and later | |
| 9807/3 | | Mixed-phenotype acute leukemia with t(v;11q23.3); KMT2A-rearranged | | 2010 and later | |
| 9808/3 | | Mixed-phenotype acute leukemia, B/myeloid, not otherwise specified | | 2010 and later | |
| 9809/3 | | Mixed-phenotype acute leukemia, T/myeloid, not otherwise specified | | 2010 and later | |
| 9823/1 | | Monoclonal B-cell lymphocytosis, CLL-type | | This neoplasm is not reportable | |
| 9591/1 | | Monoclonal B-cell lymphocytosis, non-CLL-type | | This neoplasm is not reportable | |
| 9769/1 | | Monoclonal immunoglobulin deposition disease | | This neoplasm is not reportable | |
| 9700/3 | | Mycosis fungoides | | 1978 and later | |
| 9983/3 | | Myelodysplastic syndrome with excess blasts | | 2001 and later | |
| 9986/3 | | Myelodysplastic syndrome with isolated del(5q) | | 2001 and later | |
| 9985/3 | | Myelodysplastic syndrome with multilineage dysplasia | | 2001 and later | |
| 9993/3 | | Myelodysplastic syndrome with ring sideroblasts and multilineage dysplasia | | 2021 and later | |
| 9982/3 | | Myelodysplastic syndrome with ring sideroblasts and single lineage dysplasia | | 2001 and later | |
| 9980/3 | | Myelodysplastic syndrome with single lineage dysplasia | | 2001 and later | |
| 9989/3 | | Myelodysplastic syndrome, unclassifiable | | 2001 and later | |
| 9975/3 | | Myelodysplastic/myeloproliferative neoplasm, unclassifiable | | 2010 and later | |
| 9898/3 | | Myeloid leukemia associated with Down Syndrome | | 2010 and later | |
| 9860/3 | | Myeloid leukemia, NOS | | 1978 and later | |
| 9930/3 | | Myeloid sarcoma | | 1978 and later | |
| 9967/3 | | Myeloid/lymphoid neoplasms with FGFR1 rearrangement | | 2010 and later | |
| 9968/3 | | Myeloid/lymphoid neoplasms with PCM1-JAK2 | | 2021 and later | |
| 9965/3 | | Myeloid/lymphoid neoplasms with PDGFRA rearrangement | | 2010 and later | |
| 9966/3 | | Myeloid/lymphoid neoplasms with PDGFRB rearrangement | | 2010 and later | |
| 9960/3 | | Myeloproliferative neoplasm, NOS | | 2001-2009 | |
| 9659/3 | | Nodular lymphocyte predominant Hodgkin lymphoma | | 1992 and later | |
| 9663/3 | | Nodular sclerosis classic Hodgkin lymphoma | | 1978 and later | |
| 9591/3 | | Non-Hodgkin lymphoma, NOS | | 1978 and later | |
| 9765/1 | | Non-IgM monoclonal gammopathy of undetermined significance | | This neoplasm is not reportable | |
| 9702/3 | | Peripheral T-cell lymphoma, NOS | | 1992 and later | |
| 9733/3 | | Plasma cell leukemia | | 1978-2009 | |
| 9732/3 | | Plasma cell myeloma | | 1978 and later | |
| 9735/3 | | Plasmablastic lymphoma | | 2010 and later | |
| 9950/3 | | Polycythemia vera | | 2001 and later | |
| 9971/1 | | Polymorphic post-transplant lymphoproliferative disorder | | This neoplasm is not reportable | |
| 9971/3 | | Polymorphic post-transplant lymphoproliferative disorders | | 2010-2020 | |
| 9836/3 | | Precursor B-cell lymphoblastic leukemia | | 2001-2009 | |
| 9728/3 | | Precursor B-cell lymphoblastic lymphoma | | 2001-2009 | |
| 9729/3 | | Precursor T-cell lymphoblastic lymphoma, NOS | | 1992-2009 | |
| 9835/3 | | Precursor cell lymphoblastic leukemia, NOS | | 1995-2009 | |
| 9709/1 | | Primary cutaneous CD4+ small/medium T-cell lymphoproliferative disorder | | This neoplasm is not reportable | |
| 9709/3 | | Primary cutaneous T-cell lymphoma | | 1992 and later | |
| 9718/3 | | Primary cutaneous anaplastic large cell lymphoma | | 2001 and later | |
| 9597/3 | | Primary cutaneous follicle center lymphoma | | 2010 and later | |
| 9726/3 | | Primary cutaneous gamma-delta T-cell lymphoma | | 2010 and later | |
| 9678/3 | | Primary effusion lymphoma | | 2001 and later | |
| 9679/3 | | Primary mediastinal (thymic) large B-cell lymphoma | | 2001 and later | |
| 9961/3 | | Primary myelofibrosis | | 1978 and later | |
| 9832/3 | | Prolymphocytic leukemia, NOS | | 1978 and later | |
| 9840/3 | | Pure erythroid leukemia | | 1978 and later | |
| 9984/3 | | Refractory anemia with excess blasts in transformation | | 2001-2009 | |
| 9991/3 | | Refractory neutropenia | | 2010-2020 | |
| 9992/3 | | Refractory thrombocytopenia | | 2010-2020 | |
| 9701/3 | | Sezary syndrome | | 1978 and later | |
| 9731/3 | | Solitary plasmacytoma of bone | | 1978 and later | |
| 9689/3 | | Splenic marginal zone lymphoma | | 2001 and later | |
| 9708/3 | | Subcutaneous panniculitis-like T-cell lymphoma | | 2001 and later | |
| 9724/3 | | Systemic EBV-positive T-cell lymphoma of childhood | | 2010 and later | |
| 9741/3 | | Systemic mastocytosis with an associated hematological neoplasm | | 1978 and later | |
| 9831/3 | | T-cell large granular lymphocytic leukemia | | 2010 and later | |
| 9834/3 | | T-cell prolymphocytic leukemia | | 2001 and later | |
| 9688/3 | | T-cell/histiocyte-rich large B-cell lymphoma | | 2010 and later | |
| 9768/1 | | T-gamma lymphoproliferative disorder | | This neoplasm is not reportable | |
| 9837/3 | | T-lymphoblastic leukemia/lymphoma | | 2001 and later | |
| 9987/3 | | Therapy-related myelodysplastic syndrome, NOS | | 2001-2009 | |
| 9920/3 | | Therapy-related myeloid neoplasms | | 2001 and later | |
| 9898/1 | | Transient abnormal myelopoiesis associated with Down syndrome | | This neoplasm is not reportable | |
| 9761/3 | | Waldenstrom macroglobulinemia | | 1992 and later | |

**Supplementary Table 2** BTC incidence rate by race/ethnicity: 2001–2015 [2]

|  | BTC | | GBC | | ICC | | ECC | | AVC | | Overlapping & NOS | |
| --- | --- | --- | --- | --- | --- | --- | --- | --- | --- | --- | --- | --- |
|  | IR (95% CI) | IRR (95% CI) | IR  (95% CI) | IRR (95% CI) | IR  (95% CI) | IRR (95% CI) | IR  (95% CI) | IRR (95% CI) | IR  (95% CI) | IRR (95% CI) | IR  (95% CI) | IRR (95% CI) |
| All races/ethnicities | 5.04  (5.02 to 5.06) | – | 1.57  (1.56 to 1.58) | – | 1.17  (1.16 to 1.19) | – | 1.25  (1.24 to 1.26) | – | 0.76  (0.75 to 0.77) | – | 0.29  (0.29 to 0.30) | – |
| White | 4.64  (4.62 to 4.67) | 1.0 | 1.33  (1.31 to 1.34) | 1.0 | 1.13  (1.12 to 1.15) | 1.0 | 1.19  (1.18 to 1.21) | 1.0 | 0.72  (0.71 to 0.73) | 1.0 | 0.27  (0.27 to 0.28) | 1.0 |
| Hispanic | 7.65  (7.53 to 7.76) | 1.65  (1.62 to 1.67) | 2.95  (2.88 to 3.02) | 2.22  (2.16 to 2.28) | 1.53  (1.48 to 1.58) | 1.34  (1.30 to 1.39) | 1.63  (1.57 to 1.68) | 1.36  (1.31 to 1.41) | 1.12  (1.07 to 1.16) | 1.55  (1.49 to 1.62) | 0.44  (0.42 to 0.47) | 1.62  (1.51 to 1.73) |
| Black | 5.32  (5.24 to 5.40) | 1.15  (1.13 to 1.16) | 2.19  (2.14 to 2.24) | 1.65  (1.61 to 1.69) | 1.03  (0.99 to 1.06) | 0.91  (0.87 to 0.94) | 1.15  (1.11 to 1.18) | 0.96  (0.93 to 0.99) | 0.67  (0.64 to 0.70) | 0.93  (0.89 to 0.97) | 0.29  (0.28 to 0.31) | 1.08  (1.00 to 1.15) |
| Asian or Pacific Islander | 6.74  (6.60 to 6.88) | 1.45  (1.42 to 1.48) | 1.89  (1.82 to 1.97) | 1.43  (1.37 to 1.49) | 1.63  (1.56 to 1.70) | 1.44  (1.38 to 1.50) | 1.85  (1.77 to 1.93) | 1.55  (1.48 to 1.62) | 1.03  (0.98 to 1.09) | 1.44  (1.36 to 1.52) | 0.34  (0.31 to 0.37) | 1.24  (1.12 to 1.36) |
| American Indian/Alaska Native | 5.68  (5.36 to 6.01) | 1.22  (1.15 to 1.29) | 2.57  (2.36 to 2.80) | 1.94  (1.77 to 2.11) | 1.02  (0.89 to 1.16) | 0.90  (0.79 to 1.03) | 1.18  (1.03 to 1.33) | 0.99  (0.86 to 1.12) | 0.58  (0.48 to 0.69) | 0.81  (0.67 to 0.96) | 0.33  (0.26 to 0.42) | 1.21  (0.94 to 1.54) |

IRR is calculated using White individuals as the reference group. 95% CIs which do not overlap 1 indicate statistical significance at 0.05.

*AVC* ampulla of Vater cancer, *BTC* biliary tract cancer, *CI* confidence interval, *ECC* extrahepatic cholangiocarcinoma, *GBC* gallbladder cancer, *ICC* intrahepatic cholangiocarcinoma, *IR* incidence ASR per 100,000 person-years, *IRR* incidence rate ratio with White as reference group, *NOS* not otherwise specified.

**Supplementary Table 3** Annual percent change in BTC rates: 2001–2015 [2]

| APC (95% CI) | | | | | |  |
| --- | --- | --- | --- | --- | --- | --- |
| BTC | GBC | ICC | ECC | AVC | Overlapping & NOS | |
| 1.76  (1.59 to 1.92) | –0.25  (–0.51 to 0.01) | 6.65  (6.11 to 7.19) | 0.72  (0.34 to 1.10) | –0.01  (–0.41 to -0.40) | 3.09  (1.95 to 4.25) | |

*APC* annual percent change, *AVC* ampulla of Vater cancer, *BTC* biliary tract cancer, *ECC* extrahepatic cholangiocarcinoma, *GBC* gallbladder cancer, *ICC* intrahepatic cholangiocarcinoma, *NOS* not otherwise specified.

**Supplementary Table 4** The 10-year prevalence of BTC overall and by anatomic site across different races/ethnicities in 2015^a^ [4]

|  | Prevalence rate/100,000 | | | | | |
| --- | --- | --- | --- | --- | --- | --- |
|  | BTC | GBC | ICC | ECC | AVC | Overlapping & NOS |
| All races/ethnicities | 10.8 | 3.5 | 2.3 | 2.0 | 2.8 | 0.3 |
| White | 9.4 | 2.6 | 2.2 | 1.8 | 2.5 | 0.2 |
| Hispanic | 16.6 | 6.8 | 2.6 | 2.8 | 4.1 | 0.5 |
| Black | 10.0 | 4.4 | 1.7 | 1.5 | 2.2 | 0.2 |
| Asian or Pacific Islander | 13.8 | 3.8 | 2.8 | 3.2 | 3.8 | 0.3 |
| American Indian/Alaska Native | 8.5 | 4.4 | 0.8 | 1.4 | 1.7 | 0.2 |

*AVC* ampulla of Vater cancer, *BTC* biliary tract cancer, *ECC* extrahepatic cholangiocarcinoma, *GBC* gallbladder cancer, *ICC* intrahepatic cholangiocarcinoma, *NOS* not otherwise specified.
^a^10 year age-adjusted prevalence includes patients diagnosed in the last 10 years and still alive on January 1, 2015. No 95% CI added because the SEER database does not include age-adjusted prevalence.

**Supplementary Table 5** Five-year relative survival for patients with BTC overall and by anatomic site across different races/ethnicities [5]

|  | | | Relative survival, % (95% CI) | | | | |
| --- | --- | --- | --- | --- | --- | --- | --- |
|  | BTC | GBC | | ICC | ECC | AVC | Overlapping & NOS |
| All races/ethnicities | 15.2  (14.8 to 15.7) | 18.0  (17.1 to 18.8) | | 8.5  (7.8 to 9.2) | 9.1  (8.4 to 9.8) | 34.5  (33.0 to 36.0) | 3.0  (2.2 to 4.0) |
| White | 14.8  (14.2 to 15.3) | 17.6  (16.5 to 18.7) | | 8.3  (7.2 to 9.2) | 9.3  (8.4 to 10.2) | 33.9  (31.9 to 35.8) | 2.6  (1.8 to 3.8) |
| Hispanic | 16.6  (15.4 to 17.7) | 18.7  (16.9 to 20.6) | | 7.8  (6.0 to 9.9) | 8.5  (6.7 to 10.5) | 36  (32.3 to 39.6) | 3.4  (1.5 to 6.5) |
| Black | 13.8  (12.4 to 15.2) | 16.3  (14.0 to 18.7) | | 9  (6.7 to 11.8) | 8.2  (6.1 to 10.7) | 27.2  (22.5 to 32.2) | 2.7 (0.8 to 6.8) |
| Asian or Pacific Islander | 16.5  (15.2 to 17.8) | 19.1  (16.5 to 21.8) | | 9.5  (7.5 to 11.8) | 9.6  (7.7 to 11.7) | 39.4  (34.9 to 43.8) | 5  (2.4 to 9.0) |
| American Indian/ Alaska Native | 15  (11.1 to 19.5) | 20.7  (14.2 to 28.2) | | 7.7  (2.7 to 16.1) | 5.3  (1.5 to 12.7) | 28.6  (13.1 to 46.2) | – |

Survival data are extracted as life tables from the SEER18 database [5]. The life tables are stratified by BTC type, race/ethnicity, historical stage and sex. The survival times in the lifetable are grouped into monthly intervals up to 60 months (5 years) after diagnosis. The relative survival rates, calculated as overall survival divided by the expected survival, are presented. A case lost to follow‐up is defined as “a case for which all contacts for follow‐up have been exhausted without successfully obtaining current information on the patient's health status at a point in time more than 15 months after the date of last contact”. [14]

*AVC* ampulla of Vater cancer, *BTC* biliary tract cancer, *ECC* extrahepatic cholangiocarcinoma, *GBC* gallbladder cancer, *ICC* intrahepatic cholangiocarcinoma, *NOS* not otherwise specified.
–, statistic could not be calculated.

**Supplementary Table 6** Percentage lost to follow-up by race/ethnicity and historic stage

|  | Distant | | | Localized | | | Regional | | | Unstaged | | |
| --- | --- | --- | --- | --- | --- | --- | --- | --- | --- | --- | --- | --- |
| Race/ethnicity | Patients, n | LFP, n | LFP, % | Patients, n | LFP, n | LFP, % | Patients, n | LFP, n | LFP, % | Patients, n | LFP, n | LFP, % |
| White | 8085 | 652 | 8.1 | 5394 | 960 | 17.8 | 7670 | 1108 | 14.4 | 2961 | 169 | 5.7 |
| Hispanic | 2486 | 329 | 13.2 | 1563 | 417 | 26.7 | 2026 | 450 | 22.2 | 591 | 52 | 8.8 |
| Black | 1456 | 122 | 8.4 | 835 | 203 | 24.3 | 1087 | 171 | 15.7 | 377 | 19 | 5.0 |
| Asian or Pacific Islander | 1516 | 171 | 11.3 | 1058 | 232 | 21.9 | 1593 | 328 | 20.6 | 489 | 62 | 12.7 |
| American Indian/Alaska  Native | 127 | 9 | 7.1 | 113 | 13 | 11.5 | 124 | 20 | 16.1 | 44 | 2 | 4.5 |

The original survival data for the Asian or Pacific Islander population with distant BTC and the SEER*Stat Survival Session are shown in Additional File 2.

*BTC* biliary tract cancer, *LFP* lost to follow up, *SEER* Surveillance, Epidemiology, and End Results.

**Supplementary Table 7** Original and adjusted 5-year relative survival (%) after adjusting for higher lost-to-follow-up for the race-ethnicity minority groups

|  | BTC | | GBC | | ICC | | ECC | | AVC | | Overlapping & NOS | |
| --- | --- | --- | --- | --- | --- | --- | --- | --- | --- | --- | --- | --- |
| Race/ethnicity | Orig. | Adj. | Orig. | Adj. | Orig. | Adj. | Orig. | Adj. | Orig. | Adj. | Orig. | Adj. |
| White | 14.8 | – | 17.6 | – | 8.3 | – | 9.3 | – | 33.9 | – | 2.6 | – |
| Hispanic | 16.6 | 13.6 | 18.7 | 15.6 | 7.8 | 6.1 | 8.5 | 5.8 | 36.0 | 30.5 | 3.4 | 1.4 |
| Black | 13.8 | 12.5 | 16.3 | 14.5 | 9.0 | 8.4 | 8.2 | 7.4 | 27.2 | 24.8 | 2.7 | 2.0 |
| Asian or Pacific Islander | 16.5 | 14.6 | 19.1 | 17.1 | 9.5 | 9.4 | 9.6 | 8.4 | 39.4 | 31.9 | 5.0 | 5.1 |
| American Indian/Alaska Native | 15.0 | 14.7 | 20.7 | 15.9 | 7.7 | 4.2 | 5.3 | 2.5 | 28.6 | 20.5 | – | – |

*Adj.* adjusted, *AVC* ampulla of Vater cancer, *BTC* biliary tract cancer, *ECC* extrahepatic cholangiocarcinoma, *GBC* gallbladder cancer, *ICC* intrahepatic cholangiocarcinoma, *NOS* not otherwise specified, *Orig.* original.

–, statistic could not be calculated.

**Supplementary Figures**

**Supplementary Fig. 1** Comparison of APC by sex [2]


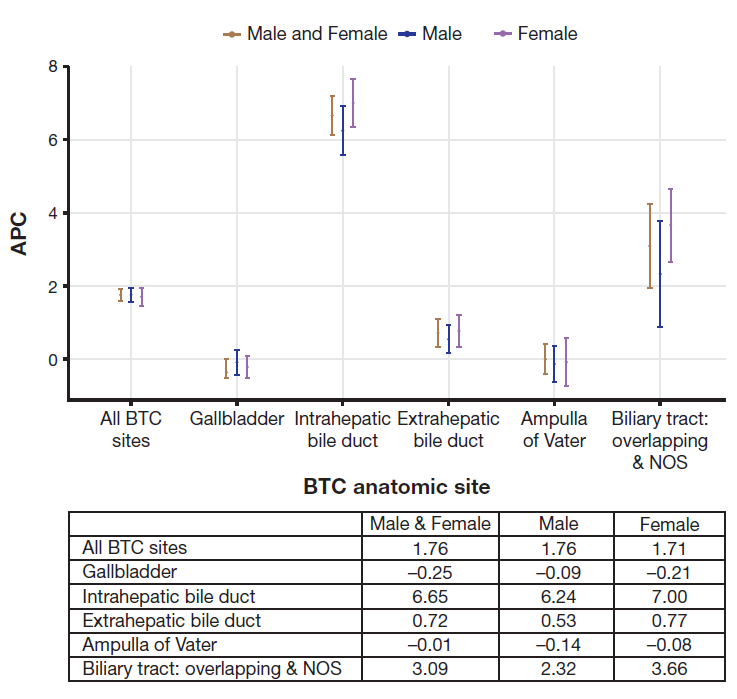


*APC* annual percent change, *BTC* biliary tract cancer, *NOS* not otherwise specified.

**Supplementary Fig. 2** Comparison of APC by race/ethnicity [2]


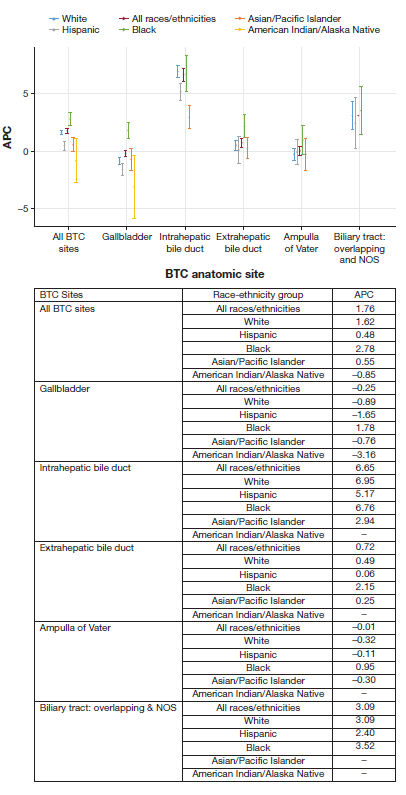


*APC* annual percent change, *BTC* biliary tract cancer, *NOS* not otherwise specified.

**Supplementary Fig. 3** Comparison of APC by age group [2]


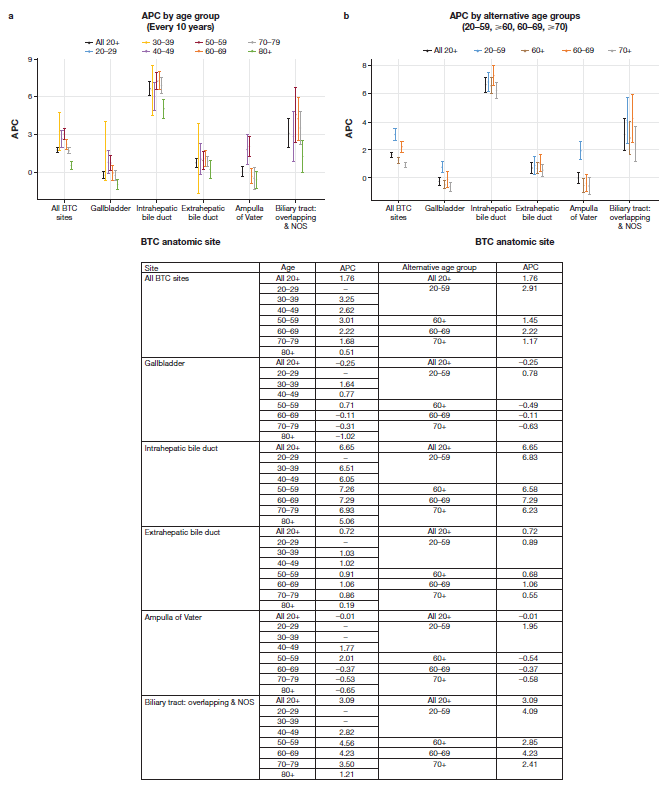


*APC* annual percent change, *BTC* biliary tract cancer, *NOS* not otherwise specified.

**Supplementary Fig. 4** BTC incidence: overlapping & NOS [2]


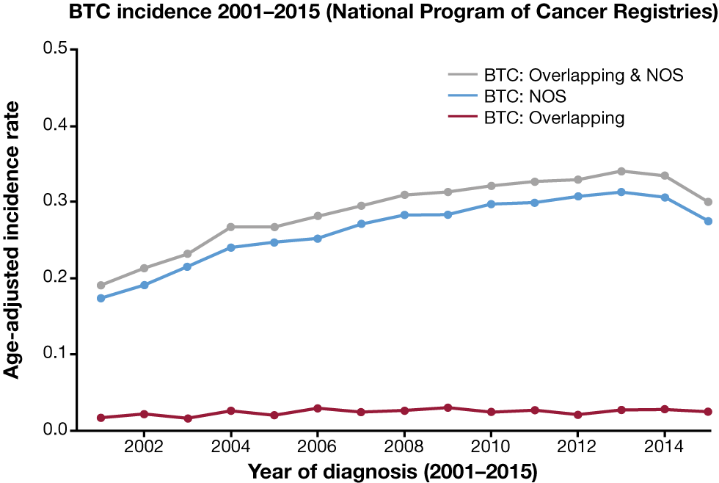


*BTC* biliary tract cancer, *NOS* not otherwise specified.

**Supplementary Fig. 5** Sensitivity analysis of extrahepatic bile duct incidence [15]


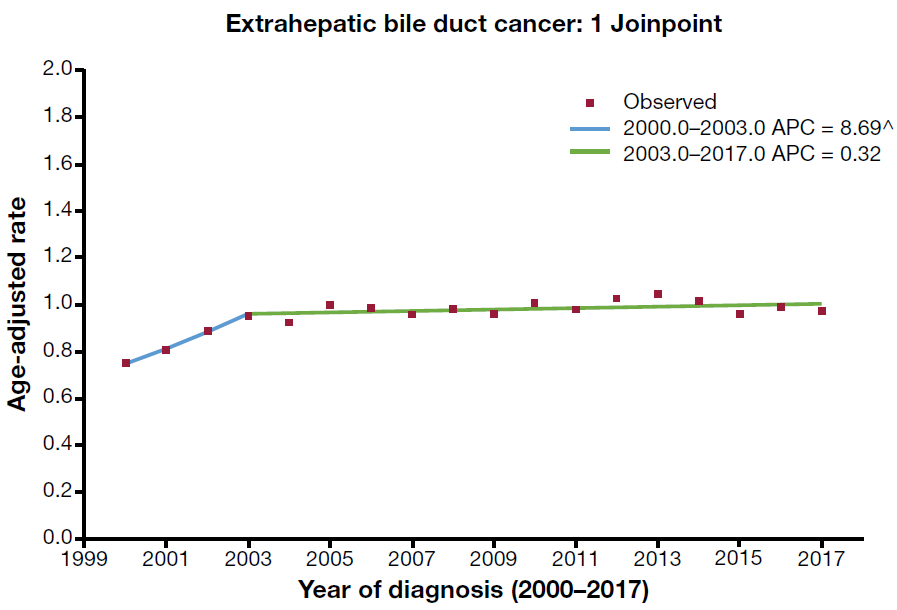


Note: ^ Indicates that the APC is significantly different from zero at the alpha = 0.05 level. *APC* annual percent change.

**Supplementary Fig. 6** Sensitivity analysis of overlapping & NOS incidence [15]


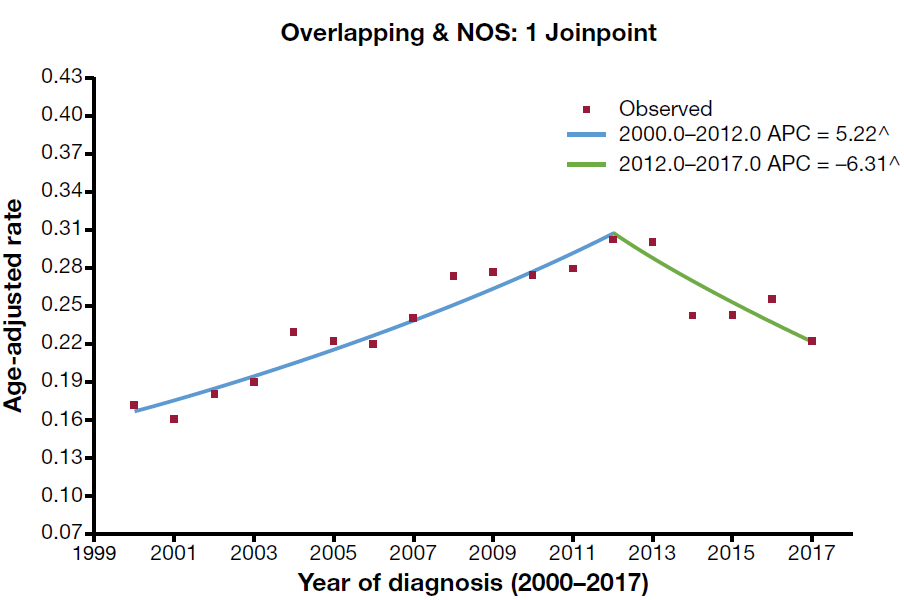


Note: ^ Indicates that the APC is significantly different from zero at the alpha = 0.05 level. *APC* annual percent change, *NOS* not otherwise specified.

**Supplementary Fig. 7** The 10-year prevalence of BTC by anatomic site and race/ethnicity in 2015^a^ [4]


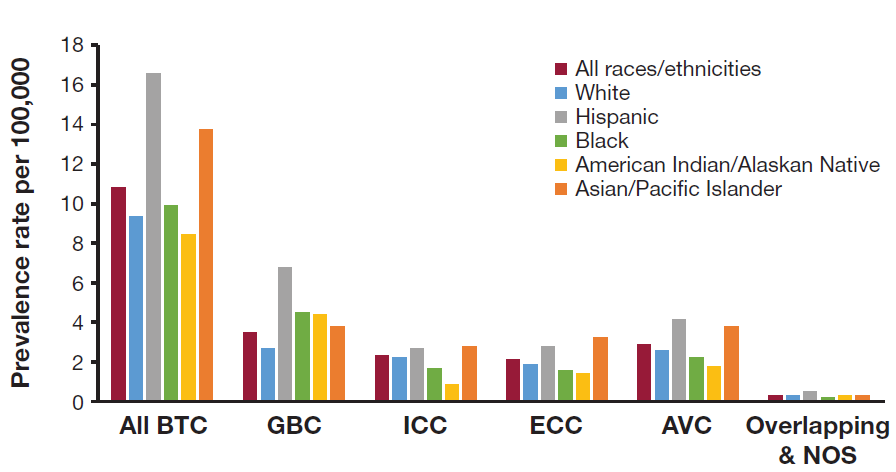


*AVC* ampulla of Vater cancer, *BTC* biliary tract cancer, *ECC* extrahepatic cholangiocarcinoma, *GBC* gallbladder cancer, *ICC* intrahepatic cholangiocarcinoma, *NOS* not otherwise specified.

^a^10-year age-adjusted prevalence includes patients diagnosed in the last 10 years and still alive on January 1, 2015.

**Supplementary Fig. 8** Mortality rate trends [2]


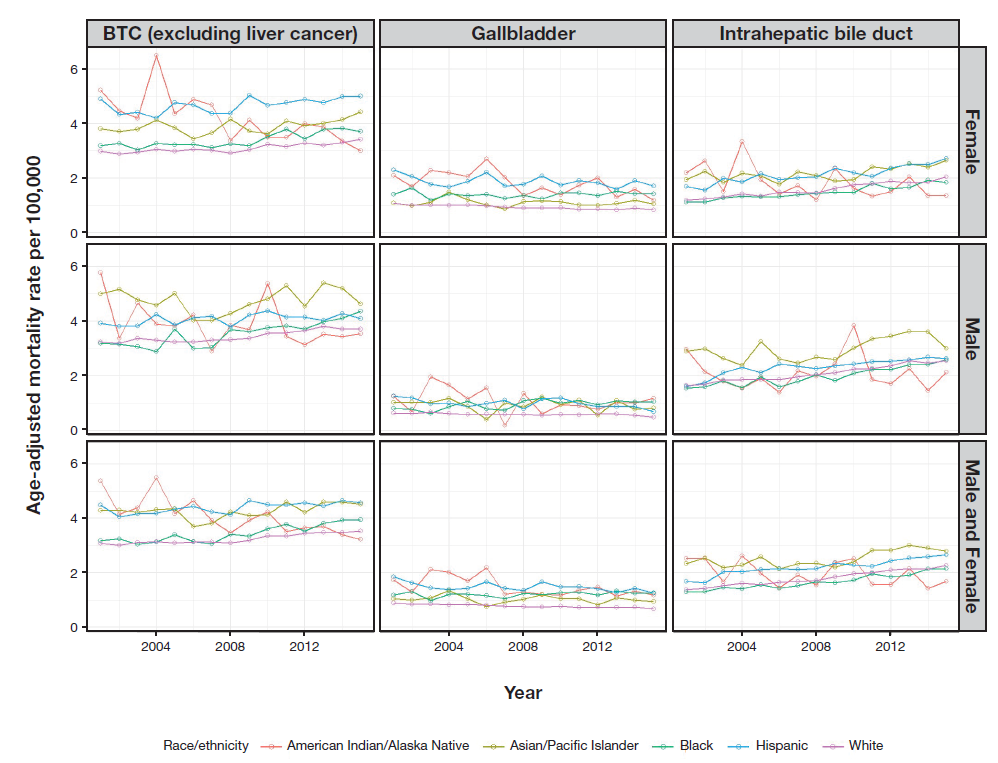


*BTC* biliary tract cancer.

**Supplementary Fig 9**

Mortality rate of BTC overall and by anatomic site in the United States: 2001–2015 [2]


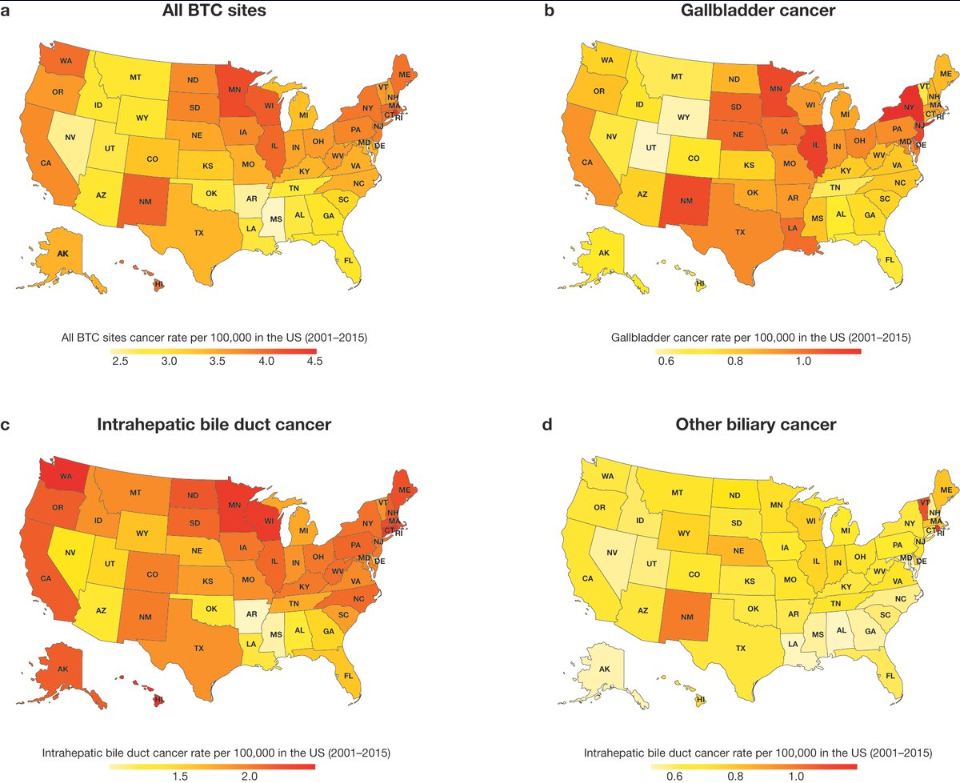


Note: Mortality rates are age-standardized and reported as cases per 100,000 person-years. Incidence-based mortality rates for ECC, AVC and Overlapping & NOS are only available for SEER 18 registries and therefore cannot be displayed completely on the US map.

*BTC* biliary tract cancer, *US* United States.

**Supplementary Fig. 10** Comparisons of relative survival between historical stages [4]


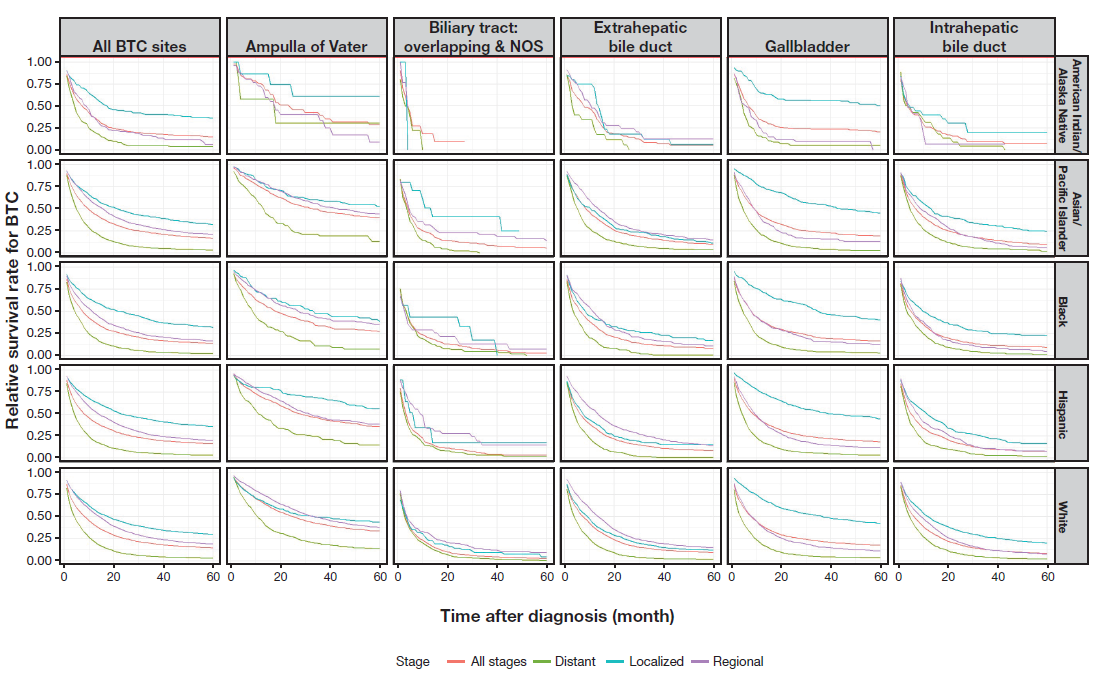


*BTC* biliary tract cancer, *NOS* not otherwise specified.

**References**

1. Centers for Disease Control and Prevention. National Program of Cancer Registries (NPCR) - about the program; 2018. Available from <https://www.cdc.gov/cancer/npcr/about.html> Accessed August 20, 2021.

2. National Program of Cancer Registries and Surveillance, Epidemiology, and End Results SEER*Stat Database: NPCR and SEER Incidence – U.S. Cancer Statistics Public Use Research Database, Nov 2017 submission (2001–2015), United States Department of Health and Human Services, Centers for Disease Control and Prevention and National Cancer Institute. Released June 2018, based on November 2017 submissions; Available from [www.cdc.gov/cancer/public-use](file:///C:\Users\Diane.Mascioli\Downloads\www.cdc.gov\cancer\public-use). Accessed September 14, 2020.

3. Surveillance, Epidemiology, and End Results (SEER) Program SEER*Stat Database: Incidence-Based Mortality - SEER 18 Regs (Excl Louisiana) Research Data, Nov 2017 Sub (2000–2015) <Katrina/Rita Population Adjustment> - Linked To County Attributes - Time Dependent (1990–2019) Income/Rurality, 1969–2020 Counties, National Cancer Institute, DCCPS, Surveillance Research Program, released April 2018, based on the November 2017 submission; Available from [www.seer.cancer.gov](C:\\Users\\Diane.Mascioli\\Downloads\\www.seer.cancer.gov). Accessed May 31, 2022.

4. Surveillance, Epidemiology, and End Results (SEER) Program SEER*Stat Database: Incidence - SEER 18 Regs Research Data + Hurricane Katrina Impacted Louisiana Cases, Nov 2017 Sub (2000-2015) <Katrina/Rita Population Adjustment> - Linked To County Attributes - Time Dependent (1990–2019) Income/Rurality, 1969–2020 Counties, National Cancer Institute, DCCPS, Surveillance Research Program, released April 2018, based on the November 2017 submission; Available from [www.seer.cancer.gov](file:///C:\Users\Diane.Mascioli\Downloads\www.seer.cancer.gov). Accessed September 14, 2020.

5. Surveillance, Epidemiology, and End Results (SEER) Program SEER*Stat Database: Incidence ‐ SEER 18 Regs Research Data + Hurricane Katrina Impacted Louisiana Cases, Nov 2017 Sub (2000‐2015) <Katrina/Rita Population Adjustment> ‐ Linked To County Attributes ‐ Total U.S., 1969‐2016 Counties, National Cancer Institute, DCCPS, Surveillance Research Program, released April 2018, based on the November 2017 submission; Available from [www.seer.cancer.gov](https://interpublic-my.sharepoint.com/personal/alex_davis_connect-cmc_com/Documents/Desktop/www.seer.cancer.gov). Accessed September 14, 2020.

6. Newsome JR, Venkatramani R, Heczey A, Danysh HE, Fishman DS, Miloh T. Cholangiocarcinoma among children and adolescents: a review of the literature and Surveillance, Epidemiology, and End Results Program database analysis. J Pediatr Gastroenterol Nutr. 2018;66(1):e12–e8.

7. World Health Organization. International classification of diseases for oncology (ICD-O). 1st revision; 3rd ed. 2013, Geneva, Switzerland: World Health Organization.

8. NIH National Cancer Institute. Surveillance Epidemiology and End Results Program. ICD code lists. Available from https://seer.cancer.gov/seertools/hemelymph/code_list/. Accessed February 11, 2021.

9. World Health Organization. Manual of the international statistical classification of diseases, injuries, and causes of death. 9th revision. 1977, Geneva, Switzerland: World Health Organization.

10. World Health Organization. Manual of the international statistical classification of diseases, injuries, and causes of death. 10th revision. 1992, Geneva, Switzerland: World Health Organization.

11. Weir HK, et al. Evaluation of North American Association of Central Cancer Registries' (NAACCR) data for use in population-based cancer survival studies. J Natl Cancer Inst Monogr. 2014;2014(49):198–209.

12. Pinheiro PS, Morris CR, Liu L, Bungum TJ, Altekruse SF. The impact of follow-up type and missed deaths on population-based cancer survival studies for Hispanics and Asians. J Natl Cancer Inst Monogr. 2014;2014(49):210–7.

13. Willems S, et al. Correcting for dependent censoring in routine outcome monitoring data by applying the inverse probability censoring weighted estimator. Stat Methods Med Res. 2018;27(2):323–35.

14. NIH National Cancer Institute. Surveillance Epidemiology and End Results Program. Follow-up process. Available from https://training.seer.cancer.gov/followup/process/. Accessed September 12, 2022.

15. Surveillance, Epidemiology, and End Results (SEER) Program SEER*Stat Database: Incidence - SEER Research Limited-Field Data, 21 Registries, Nov 2019 Sub (2000–2017) - Linked To County Attributes - Time Dependent (1990–2017) Income/Rurality, 1969–2018 Counties, National Cancer Institute, DCCPS, Surveillance Research Program, released April 2020, based on the November 2019 submission; Available from [www.seer.cancer.gov](file:///C:\Users\Diane.Mascioli\Downloads\www.seer.cancer.gov). Acessed January 12, 2021.
